# Bioadhesive Hydrogel With Polyphenol‐Armored Nanogene Rejuvenates Chondrocyte Senescence for Aged Osteoarthritis Therapy

**DOI:** 10.1002/advs.202600014

**Published:** 2026-05-20

**Authors:** Liwei Yan, Runze Yang, Ting Zhou, Tianhao Xu, Yongqi Li, Yuelin Hu, Minghao Ge, Lei Zhang, Xiong Lu, Chaoming Xie, Weili Fu

**Affiliations:** ^1^ Sports Medicine Center Department of Orthopedic Surgery/Orthopedic Research Institute West China Hospital Sichuan University Chengdu Sichuan China; ^2^ Institute of Biomedical Engineering College of Medicine Southwest Jiaotong University Chengdu China; ^3^ Department of Orthopedics Sichuan Provincial People's Hospital University of Electronic Science and Technology of China Chengdu Sichuan China; ^4^ National Engineering Research Center for Biomaterials College of Biomedical Engineering Sichuan University Chengdu Sichuan China

**Keywords:** adhesive hydrogels, aging, gene delivery, osteoarthritis, polyphenol

## Abstract

Chondrocyte senescence, exacerbated by ageing and cellular stress, is a key driver of metabolic imbalance during aged osteoarthritis (OA) progression. Achieving sustained inhibition of chondrocyte senescence while mitigating multifactorial cellular stressors remains challenging in aged OA treatment. In this study, an injectable bioadhesive and lubricating hydrogel, encapsulating miR‐140‐5p (miR‐140)‐loaded and polyphenol‐armored nanoparticles, was developed for rejuvenating senescent chondrocytes in aged OA. Originating from catechol groups, the hydrogel anchors firmly to cartilage tissues, facilitating a sustained reduction in joint friction. It also acts as a local depot for nanoparticles, prolonging their retention within the harsh joint cavity. The polyphenol armor on the nanoparticles preserves miR‐140 activity in the RNase‐ and ROS‐rich senescence microenvironment, prevents premature leakage, and enhances transfection efficiency by overcoming extracellular matrix, cell membrane, and lysosomal barriers. This contributes to the downregulation of senescence‐associated signaling pathways in chondrocytes. Furthermore, the polyphenol armor exhibits catalase‐ and superoxide‐dismutase‐like activity, mitigating mitochondrial dysfunction through targeted ROS scavenging. By integrating these advanced attributes, the hydrogel attenuated chondrocyte senescence and OA progression in an aged rat model, showing great prospects in clinical application.

## Graphical abstract

An adhesive hydrogel depot with polyphenol‐armored nanogenes is developed for treating aged osteoarthritis. This system maintains miR‐140 bioactivity, prolongs its intra‐articular retention, and enhances transfection into chondrocytes, leading to the downregulation of senescence‐associated pathways. It also mitigates mechanical and oxidative stress through lubricating effects and mitochondria‐targeted reactive oxygen species scavenging, thereby reducing key drivers of cellular senescence.

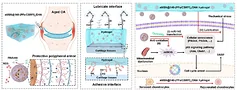

## 1 Introduction

Osteoarthritis (OA) is an age‐driven degenerative joint disease with a markedly increased prevalence in individuals over 65 years of age [1, 2]. It poses significant challenges to personal health, along with socioeconomic burdens. Chondrocyte senescence is a distinctive feature in aged OA and is recognized as a critical risk factor for disease development [3, 4, 5]. Senescent chondrocytes exhibit several hallmarks, including telomere erosion, dysregulation of signaling molecules dysregulation, mitochondrial dysfunction‐induced reactive oxygen species (ROS) accumulation, and overproduction of senescence‐associated secretory phenotype (SASP) factors—particularly proinflammatory cytokines, chemokines, and proteases [1, 6]. These products create a senescence microenvironment (SME) that shifts cartilage homeostasis from anabolic to catabolic outcomes, ultimately leading to progressive degeneration of articular cartilage with compromised joint functions [7]. Accumulating evidence indicates that miR‐140 plays an essential regulatory role in maintaining cartilage homeostasis. Specifically, miR‐140 targets histone deacetylase (HDAC)‐4 and mothers against decapentaplegic homolog (Smad) to inhibit chondrocyte hypertrophy [8]. It also acts on insulin‐like growth factor binding protein (IGFBP)‐5 to suppress extracellular matrix degradation [9]. Particularly, miR‐140 effectively inhibits chondrocyte senescence by downregulating the PI3K‐p53 signaling axis [10, 11]. Notably, miR‐140 expression is significantly downregulated in patients with aged OA [12]. While exogenous miR‐140 administration has therapeutic potential for mitigating chondrocyte senescence, its clinical efficacy is significantly limited by inherent delivery challenges. These include susceptibility to degradation in RNase‐ and ROS‐enriched SME [3, 13], rapid clearance by the intra‐articular vascular and lymphatic systems [14, 15], and poor bioavailability because of the biological barriers of negatively charged cartilage extracellular matrix (ECM), cell membranes, and lysosomes [16, 17]. These challenges underscore the need to develop an arthritis‐tailored gene delivery platform that integrates superior transfection efficacy with sustained therapeutic duration to suppress chondrocyte senescence.

Injectable hydrogels represent a promising gene delivery platform for OA treatment because of their biocompatibility, tunable functionality, and minimally invasive administration [18, 19, 20]. Furthermore, their macroscale dimensions help restrict intra‐articular clearance, prolonging retention of delivered drugs [21]. However, most hydrogel‐based gene delivery platforms developed have two critical limitations. First, hydrogels lack sufficient bioadhesiveness to anchor stably to cartilage surfaces, compromising their prolonged retention within the joint cavity [22]. While increasing the injection frequency can extend the therapeutic duration, it also increases the risk of infection and patient psychological resistance. Second, hydrogels exhibit inadequate targeting specificity and fail to provide comprehensive protection against RNase degradation and ROS damage within the SME, during the critical window from gene release to transfection. Integrating nanocarriers into hydrogel matrices is a viable solution that combines the precise delivery advantages of nanocarriers with the sustained retention benefits of hydrogels [20]. Nevertheless, this prolonged retention commonly results in the functional failure of nanocarriers. This is because miRNA drugs are commonly hydrophilic [23], which tend to diffuse prematurely from nanocarriers into the hydrogel matrix or SME. In addition, current strategies primarily focus on anti‐senescence through genetic regulation, overlooking critical persistent drivers of chondrocyte senescence in aged OA, such as impaired joint lubrication [24, 25], and mitochondrial dysfunction [26, 27]. Therefore, it is necessary to develop hydrogel‐based systems that integrate enhanced tissue adhesion and lubrication, precision and extended gene delivery capability, and mitochondrial ROS (mtROS) scavenging capabilities for the comprehensive management of aged OA.

Polyphenols, which are characterized by highly reactive catechol groups, make multiple bindings to biological tissues possible and are widely employed as adhesives in biomedical applications [28, 29]. Polyphenols can also chelate with iron ions to form redox pairs that exhibit enhanced antioxidative enzyme‐like activity [30, 31, 32], such as catalase (CAT) and superoxide dismutase (SOD). Inspired by polyphenol chemistry, this study developed an injectable, bioadhesive, and lubricating hydrogel, encapsulating polyphenol‐armored and miR‐140‐5p (miR‐140)‐loaded nanoparticles, to mitigate chondrocyte senescence in aged OA. Originating from catechol groups, the hydrogel demonstrated enhanced bioadhesion while maintaining lubricating performance, thereby reliably reducing joint friction. The polyphenol armor was engineered to confer a physical barrier with a positively charged surface and antioxidative enzyme‐like activity, which preserved miR‐140 activity, promoted gene transfection efficiency, and restored mitochondrial dysfunction. Consequently, the hydrogel significantly attenuated chondrocyte senescence and promoted cartilage anabolism, suppressing OA progression in aged rat models.

## 2 Results and Discussion

### 2.1 Design Strategy

**FIGURE 1:**
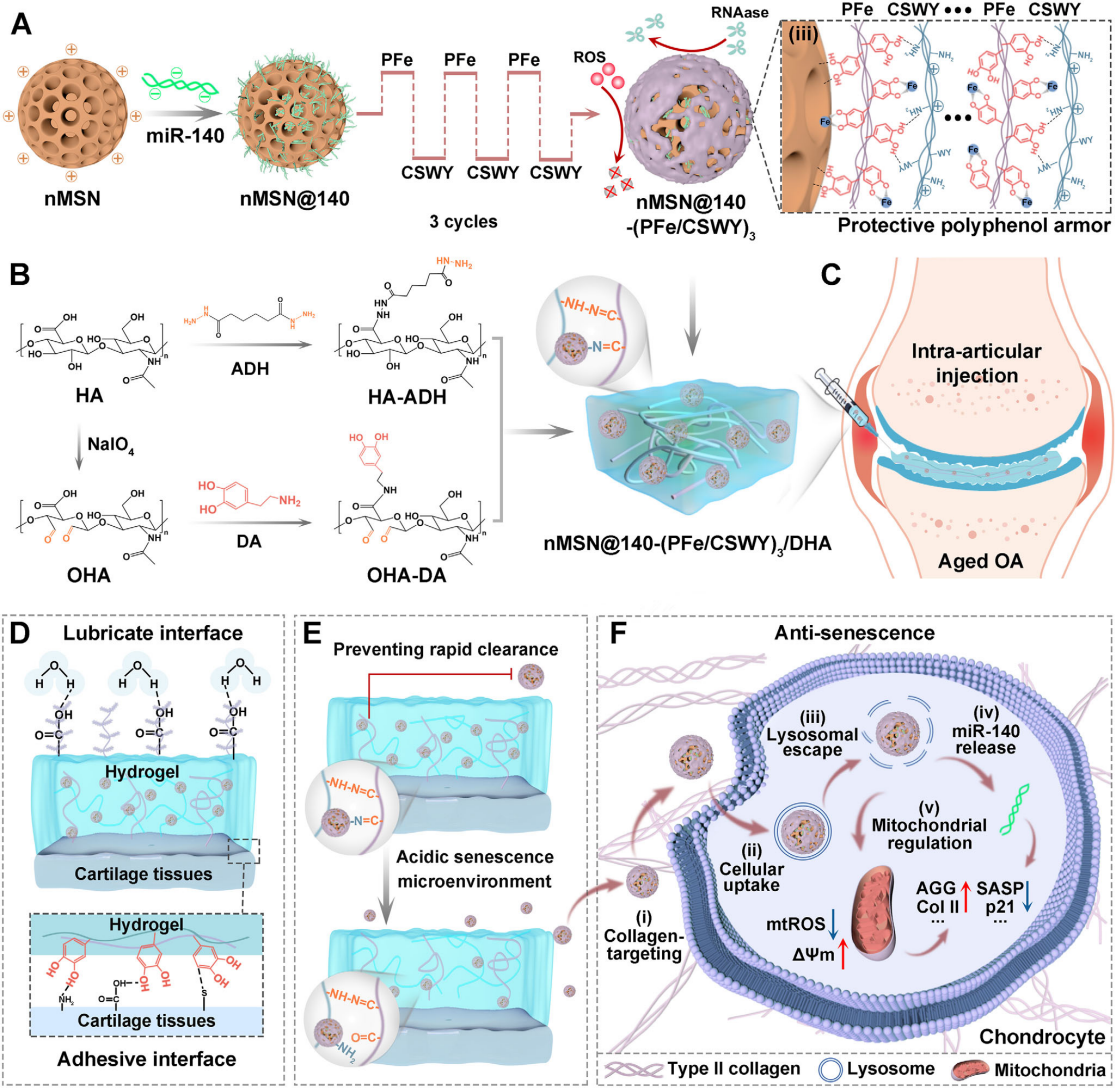
Schematic diagram of the adhesive and lubricating hydrogel with encapsulation of polyphenol‐armored nanoparticles for rejuvenating chondrocyte senescence in aged OA. (A) Illustration of the preparation of polyphenol‐armored nanoparticles through the layer‐by‐layer method. The armor is capable of physically shielding RNase and chemically eliminating ROS for miR‐140 protection. (B) Formation of the nanoparticle‐incorporated hydrogel by dynamic acylhydrazone and imine bonds. (C) In situ gelation of the hydrogel after intra‐articular injection into the OA joint cavity. (D) The hydrogel adheres to cartilage tissues and provides reliable lubrication. (E) The hydrogel serves as a depot of nanoparticles to prolong their retention time and dynamically releases nanoparticles in response to the acidic SME. (F) The nanoparticles released from the hydrogel can (i) target the cartilage ECM, (ii) internalize into chondrocytes, (iii) escape from lysosomes, (iv) promote miR‐140 transfection, and (v) mitigate mitochondrial dysfunction, contributing to rejuvenating chondrocyte senescence.

Polyphenol‐armored nMSN@140‐(PFe/CSWY)_3_ nanoparticles are synthesized through a layer‐by‐layer self‐assembly method (Figure 1A). Positively charged amino mesoporous silica nanoparticles (nMSN) are selected as carriers to enable electrostatic packaging of miR‐140. A protective polyphenol armor is then covered on the gene‐loaded nanoparticles by alternately depositing a polydopamine‐iron (PFe) layer and a type II collagen‐targeting WYRGRL peptide‐modified chitosan (CSWY) layer for several cycles. During each cycle, dopamine monomers are chelated with iron ions, anchored on the nMSN or the previous layer, and polymerized in an alkali condition to form the PFe layer. Furthermore, the catechol groups in the PFe layer provide binding sites to attract CSWY, driving the formation of the CSWY layer. By adjusting the number of deposition cycles, the polyphenol armor can serve as a barrier to prevent miR‐140 leakage and physically shield RNase. Moreover, the polyphenol‐armored nanoparticles can eliminate ROS owing to the antioxidative enzyme‐like activity of the catechol/quinone‐iron redox pairs in the PFe layer, while exhibiting a positively charged surface resulting from the amino groups in the outermost CSWY layer.

The nMSN@140‐(PFe/CSWY)_3_/DHA hydrogel is fabricated by incorporating the polyphenol‐armored nanoparticles into a dynamically crosslinked polymer matrix composed of dopamine‐modified oxidized hyaluronic acid (OHA‐DA) and adipic acid dihydrazide‐modified hyaluronic acid (HA‐ADH) (Figure 1B,C). The acylhydrazone linkages between the aldehyde groups in OHA‐DA and the hydrazide groups in HA‐ADH allow a sol–gel transition, endowing the hydrogel with injectability. The aldehyde groups in OHA‐DA are also linked to the amino groups on the polyphenol‐armored nanoparticles through pH‐sensitive imine bonds. The catechol groups in OHA‐DA and nanoparticles offer interfacial binding sites to cartilage tissues, whereas the carboxyl groups in the HA backbone act as lubricants by generating a hydration layer.

The action mechanism of the hydrogel in the treatment of aged OA is summarized as follows. First, the hydrogel with adhesive catechol groups and lubricating carboxyl groups can be anchored in situ on cartilage tissues after intra‐articular injection, while providing reliable lubrication to reduce joint friction (Figure 1D). Second, the hydrogel serves as a local depot of polyphenol‐armored nanoparticles to prolong their retention in the harsh joint cavity, which dynamically releases the nanoparticles in response to the acidic SME (Figure 1E). Third, the nanoparticles with WY peptide segments can actively target type II collagen in the cartilage ECM. Meanwhile, the polyphenol armor reduces the premature leakage of miR‐140 and protects its activity against RNase degradation and ROS damage in the SME. Subsequently, the polyphenol‐armored nanoparticles with positively charged surfaces can be internalized into chondrocytes by crossing the barriers of the cell membrane and lysosomes, promoting the transfection of miR‐140. Meanwhile, the redox catechol/quinone‐iron pairs in armor eliminate the excessive mtROS to restore mitochondrial dysfunction, synergistically contributing to the inhibition of chondrocyte senescence (Figure 1F).

### 2.2 Characterization of the Polyphenol‐Armored Nanoparticles

The synthesis of the CSWY was first characterized using ^1^H nuclear magnetic resonance (^1^H NMR) spectroscopy. Compared with unmodified chitosan (CS), the spectrum of CSWY at 3.0–3.9 ppm changed due to the peptide modification (Figure S1A,B). The grafting ratio was further evaluated using a bicinchoninic acid (BCA) assay. According to the WYRGRL standard curve (Figure S1C), the grafting ratio of CSWY was quantified to be 18.9 ± 8.9 mg/g.

**FIGURE 2:**
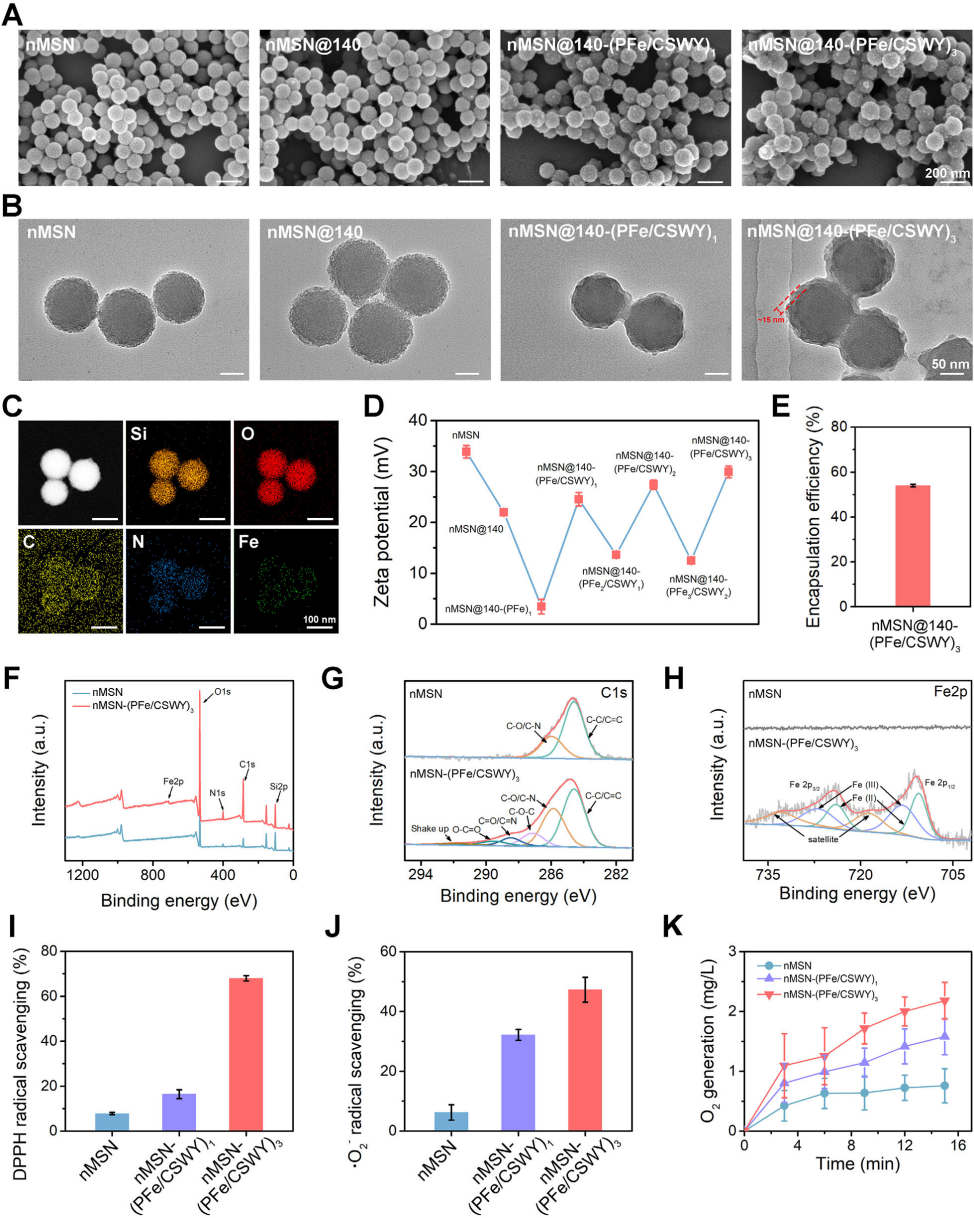
Characterization of the polyphenol‐armored nanoparticles. (A) SEM and (B) TEM images of the nMSN, nMSN@140, nMSN@140‐(PFe/CSWY)_1_, and nMSN@140‐(PFe/CSWY)_3_ nanoparticles. (C) EDS mapping images showing the elemental distribution of the nMSN@140‐(PFe/CSWY)_3_ nanoparticles. (D) Zeta potentials of the nanoparticles at each step of the synthesis of nMSN@140‐(PFe/CSWY)_3_. (E) Encapsulation efficiency of the nMSN@140‐(PFe/CSWY)_3_ nanoparticles for FAM‐labeled miR‐140. (F) XPS survey spectra, (G) C 1s high‐resolution spectra, and (H) Fe 2p high‐resolution spectra of the nMSN and nMSN@140‐(PFe/CSWY)_3_ nanoparticles. (I) DPPH radical and (J) superoxide radical scavenging efficiencies of the nanoparticles with different deposition cycles of polyphenol armor. (K) Oxygen generation converted from H_2_O_2_ by the nanoparticles with different deposition cycles of polyphenol armor.

The morphology of the nMSN@140‐(PFe/CSWY)_3_ nanoparticles was visualized using scanning electron microscopy (SEM) and transmission electron microscopy (TEM) (Figure 2A,B). The gene‐loaded nMSN@140 nanoparticles presented a spherical morphology with a diameter of approximately 100 nm, consistent with the pristine nMSN nanoparticles. A core–shell morphology was clearly observed after the deposition of the PFe and CSWY layers on the nanoparticles. The thickness of the coating increased with the number of deposition cycles, reaching approximately 15 nm for the nMSN@140‐(PFe/CSWY)_3_ nanoparticles. Energy dispersive spectroscopy (EDS) results indicated the colocalization of Si, O, C, N, and Fe signals (Figure 2C), suggesting the deposition of polyphenol armor on the nanoparticles.

The layer‐by‐layer self‐assembly process was verified using zeta potential analysis (Figure 2D). The nMSN nanoparticles exhibited a positive charge (33.9 ± 1.2 mV) in deionized water, and it became less positive (22.0 ± 0.3 mV) after packaging of miR‐140. The subsequent deposition of the polyphenol armor resulted in an alternate variation in the surface charge, which corresponded to the PFe and CSWY layers. The resulting nMSN@140‐(PFe/CSWY)_3_ nanoparticles had a positive charge of 30.0 ± 1.2 mV. The encapsulation efficiency of the nanoparticles for FAM‐labeled miR‐140 was calculated to be 54.0% ± 0.5%, as determined using a fluorescence spectrophotometer (Figure 2E). The chemical state was investigated by X‐ray photon–electron spectroscopy (Figure 2F). The nMSN@140‐(PFe/CSWY)_3_ nanoparticles possessed a higher proportion of C and Fe atoms than the nMSN (Table S1). The high‐resolution C1s spectrum indicated that the nMSN@140‐(PFe/CSWY)_3_ nanoparticles presented new peaks at 287.0, 288.3, 289.3, and 291.5 eV, corresponding to C─O─C, C═O/C═N, O─C═O, and shake‐up, respectively (Figure 2G). These peaks correspond to glycosidic bonds in the chitosan backbone of the CSWY layer and catechol/quinone groups in the polydopamine of the PFe layer. The high‐resolution profile of Fe2p indicated the presence of divalent and trivalent states of iron in the nMSN@140‐(PFe/CSWY)_3_ nanoparticles (Figure 2H). Direct characterization of chelation between PDA and iron ions was evaluated by UV–vis spectroscopy. The PDA exhibited a characteristic peak at 280 nm, which is associated with the π→π^*^ electronic transition within the catechol groups. After the incorporation of iron ions, the peak of PFe shifted to 282 nm. Moreover, two distinct new bands located at 424 and 725 nm emerged for PFe (Figure S2). These bands correspond to ligand‐to‐metal charge transitions, evidently suggesting the chelation between polydopamine and iron ions. These results confirm the synthesis of nMSN@140‐(PFe/CSWY)_3_ nanoparticles with a positively charged polyphenol armor.

The release of miR‐140 from polyphenol‐armored nanoparticles was investigated in phosphate‐buffered saline (PBS) at pH 6.5, which mimics the acidic SME [33]. The results show that the naked nMSN@140 nanoparticles exhibited a burst release behavior, with approximately 84.3% ± 2.8% (1.7 ± 0.1 µg/g) of miR‐140 released over 168 h. By contrast, the polyphenol armor on the nanoparticles significantly retarded miR‐140 release, with only 15.0% ± 1.0% (0.3 ± 0.0 µg/g) of the preloaded miR‐140 being released from the nMSN@140‐(PFe/CSWY)_3_ nanoparticles over the same period (Figure S3). Furthermore, the polyphenol armor nearly decomposed after 4 h of immersion in PBS at pH 4.5 (Figure S4), a microenvironment in lysosomes [34]. These results reveal that the polyphenol armor reduced the leakage of miR‐140 from the nanoparticles in the SME and was capable of releasing miR‐140 after lysosomal escape.

### 2.3 Antioxidative Enzyme‐Like Capacity of the Polyphenol‐Armor Nanoparticles

The polyphenol‐armored nanoparticles possessed antioxidative enzyme‐like activity that can eliminate excessive ROS and H_2_O_2_. The antioxidative enzyme‐like activity of nanoparticles was first evaluated using a representative 1‐diphenyl‐2‐picrylhydrazyl (DPPH) radical model. As shown in Figure 2I, the nMSN eliminated only 7.8% ± 0.5% of DPPH radicals after 3 h of incubation. The scavenging efficiency was dramatically improved after the deposition of polyphenol armor, in which the nMSN‐(PFe/CSWY)_3_ nanoparticles (68.0% ± 1.2%) achieved higher DPPH radical scavenging efficiency than that of the nMSN‐(PFe/CSWY)_1_ nanoparticles (16.5% ± 2.0%). Next, the ability of the nanoparticles to eliminate superoxide radicals (•O_2_^−^), a typical ROS in mitochondria, was explored using an assay kit (Figure 2J). The result indicates that the nMSN‐(PFe/CSWY)_3_ nanoparticles possessed SOD‐like activity, which eliminated 47.3% ± 4.2% •O_2_^−^ and had higher scavenging efficiency than that of the nMSN (6.2% ± 2.6%) and the nMSN‐(PFe/CSWY)_1_ nanoparticles (32.2% ± 1.8%). In addition, the CAT‐like activity of nanoparticles was evaluated by detecting the generation of oxygen converted from hydrogen peroxide (Figure 2K). The nMSN‐(PFe/CSWY)_3_ nanoparticles generated 2.2 ± 0.3 mg/L of O_2_ after 15 min of reaction with H_2_O_2_. By contrast, only 0.8 ± 0.3 mg/L of O_2_ was generated in nMSN suspension. These results indicate the remarkable ROS and H_2_O_2_ scavenging ability of the polyphenol‐armored nMSN‐(PFe/CSWY)_3_ nanoparticles.

To verify which specific component is responsible for the catalytic activity, the individual components of the polyphenol‐armored nanoparticles were evaluated for their CAT‐ and SOD‐like activities (Figure S5). The naked nMSN nanoparticles and CSWY showed only 8.3% ± 10.1% and 7.8% ± 5.1% superoxide radical scavenging efficiencies, respectively, suggesting negligible SOD‐like activity for both. By contrast, PDA, Fe, and PFe exhibited significant SOD‐like activity, among which the PFe (86.0% ± 9.2%) demonstrated a higher radical scavenging efficiency compared to PDA (74.9% ± 5.4%) or Fe (70.7% ± 3.4%). Furthermore, nMSN, PDA, and CSWY showed 2.8 ± 0.2, 2.3 ± 0.6, and 2.5 ± 0.0 mg/L of oxygen generation, respectively. These values are comparable to the background self‐decomposition of H_2_O_2_, demonstrating no CAT‐like activity of these components. However, PFe generated 16.6 ± 1.1 mg/L of oxygen, which was also higher than that of Fe (9.3 ± 1.2 mg/L). These results indicated that the PFe component in polyphenol armor plays the dominant role in SOD‐ and CAT‐like activities, which can be attributed to a dual mechanism. First, iron possesses CAT‐ and SOD‐like activities that can catalyze ROS or H_2_O_2_ into nontoxic H_2_O and O_2_ through a reversible valence transition. Second, the catechol or quinone groups can provide or receive electrons to or from iron, accelerating the valence transition of iron and thus enhancing the catalytic efficiency [22].

### 2.4 Injectability of the Nanoparticle‐Incorporated Hydrogel

The ^1^H NMR spectra of HA, OHA, OHA‐DA, and HA‐ADH are presented in Figure S6. The characteristic signal at 1.9 ppm, corresponding to the N‐acetylmethyl protons, was well‐preserved across all HA‐based spectra. In the OHA and OHA‐DA spectra, new signals appeared at 4.9–5.2 ppm, which correspond to the hemiacetal protons formed after oxidation. Furthermore, the OHA‐DA spectrum exhibited distinct peaks at 6.6–6.8 ppm, attributed to the aromatic protons of the conjugated dopamine, thereby confirming the oxidation and dopamine grafting. Based on the ^1^H NMR integration, the oxidation degree and the dopamine grafting ratio of OHA‐DA were calculated to be 21% and 3%, respectively. For HA‐ADH, new signals corresponding to the methylene protons of the adipic dihydrazide linker were observed at 1.5–1.6 and 2.1‐2.4 ppm, with a calculated grafting ratio of 26%.

**FIGURE 3:**
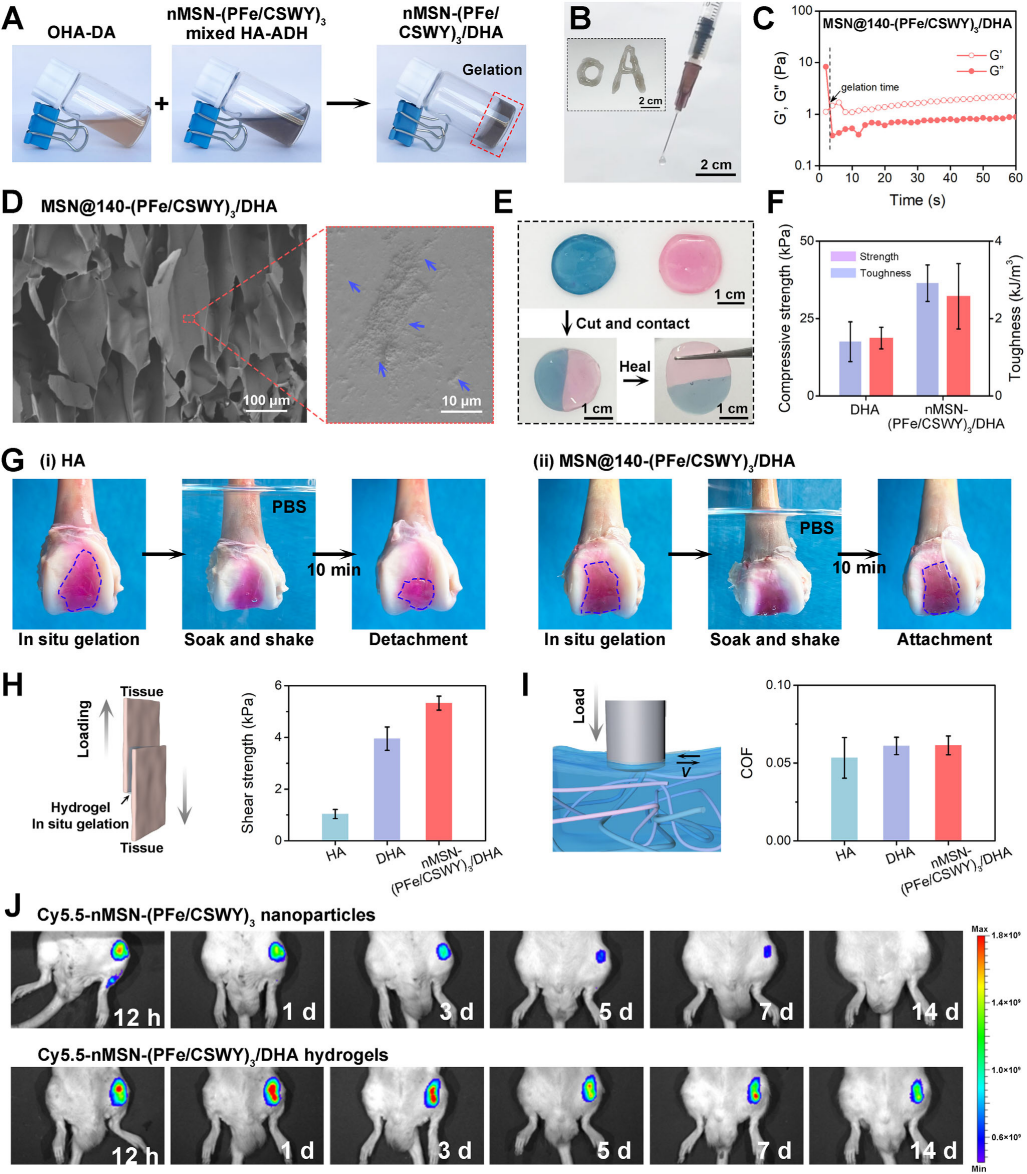
Injectability, tissue adhesion, and lubricating performances of the nanogene‐incorporated hydrogel. (A) Formation of the nMSN‐(PFe/CSWY)_3_/DHA hydrogel in a tilted bottle. (B) The hydrogel precursor was injected from a 28‐G syringe needle and gelled to form the letters “OA.” (C) Rheological time sweep of the nMSN‐(PFe/CSWY)_3_/DHA hydrogel under a strain of 1% and frequency of 1 Hz. (D) SEM images of the nMSN@140‐(PFe/CSWY)_3_/DHA hydrogel. Blue arrows indicate the nanoparticles. (E) Digital images indicating the self‐healing behaviors of the hydrogels. (F) Compressive strength and toughness of the hydrogels. (G) Digital images indicating the reliable adhesion of nMSN‐(PFe/CSWY)_3_/DHA hydrogel to rat cartilage. (H) Lap‐shear adhesion strengths of the hydrogels. (I) COFs of the hydrogels in PBS. (J) Live animal imaging of the Cy5.5‐labeled nanoparticles, with or without hydrogel encapsulation, in rat joint cavities.

The nMSN‐(PFe/CSWY)_3_/DHA hydrogel was formed by mixing the nanoparticles, OHA‐DA, and HA‐ADH polymer solution, as indicated by the sol–gel transition observed in a tilted bottle (Figure 3A). The injectability of the nMSN‐(PFe/CSWY)_3_/DHA hydrogel was verified using a 26‐G needle syringe. As shown in Figure 3B, the mixed precursor solution could be pushed out through the needle and subsequently gelled to form the letters “OA.” These results demonstrate that the hydrogel can be administered via minimally invasive intra‐articular injection and can rapidly solidify in situ to prevent joint leakage, which are essential properties for OA therapy. Rheological results indicate that the storage modulus (*G*′) and loss modulus (*G*′′) of the nMSN‐(PFe/CSWY)_3_/DHA hydrogel intersect within 4 s, indicating the gelation time of the hydrogel (Figure 3C). The nMSN‐(PFe/CSWY)_3_/DHA hydrogel had a porous structure with numerous nanoparticles covering the wall surface, in sharp contrast to the smooth wall surface of the DHA hydrogel (Figure 3D and Figure S7). EDS mapping results indicate the presence of Si and Fe signals at the cross‐section of the hydrogel (Figure S8), implying the encapsulation of nanoparticles in the hydrogels.

### 2.5 Mechanical Performance of the Hydrogel

The nMSN‐(PFe/CSWY)_3_/DHA hydrogel with dynamic linkages displayed self‐healing performance, which is conducive to withstanding repeated loading in the joint cavity. As illustrated in Figure 3E, two pieces of the nMSN‐(PFe/CSWY)_3_/DHA hydrogel, one stained with rhodamine and the other stained with trypan blue, were fractured and then contacted at the cross‐section. After 1 h of storage at 37°C, the separated pieces were integrated into a single gel. The rheological tests showed that the hydrogel ruptured (*G*′ < *G*′′) at a higher strain (300%) and then recovered (*G*′ > *G*′′) within seconds at 1% strain (Figure S9), confirming the self‐healing behavior of the hydrogel owing to the recovery of the dynamic acylhydrazone linkages. Figure 3F shows the compressive properties of the hydrogels. The nMSN‐(PFe/CSWY)_3_/DHA hydrogel exhibited a compressive strength of 36.4 ± 5.9 kPa and a toughness of 2.6 ± 0.8 kJ/m^3^, which are 2.1 and 1.7 times those of the pure DHA hydrogel, respectively. This enhancement is attributed to the nano‐reinforcement effect resulting from the incorporation of nanoparticles.

### 2.6 Adhesive and Lubricating Performances of the Hydrogel

The in situ formed nMSN‐(PFe/CSWY)_3_/DHA hydrogel could tightly adhere to cartilage tissues after intra‐articular injection while providing lubrication. As shown in Figure 3G, the nMSN‐(PFe/CSWY)_3_/DHA hydrogel was evenly distributed on the isolated joint tissue and maintained attachment even after immersion in PBS for 10 min; however, the HA hydrogel, composed of OHA and HA‐ADH without catechol groups, was partially separated from the cartilage tissues. The adhesion strength of the hydrogels to tissues was measured using a lap‐shear test. Porcine skin was selected as the representative tissue. The nMSN‐(PFe/CSWY)_3_/DHA hydrogels exhibited 5.3 ± 0.3 kPa of shear strength, which was 1.3‐ and 5.1‐fold higher than those of the DHA and HA hydrogels, respectively (Figure 3H). Furthermore, the interfacial toughness of the HA, DHA, and nMSN‐(PFe/CSWY)_3_/DHA hydrogel was 8.8 ± 1.6, 15.0 ± 4.2, and 22.0 ± 3.1 J/m^2^, respectively (Figure S10), as determined via a 180° peeling test. These results indicate the enhanced adhesion of the nMSN‐(PFe/CSWY)_3_/DHA hydrogel, originating from the catechol groups in the nanoparticles and OHA‐DA polymer chains. The lubricating performance of the hydrogels was assessed using a tribological test with a load of 1 N and a sliding frequency of 1 Hz (Figure 3I). The nMSN‐(PFe/CSWY)_3_/DHA hydrogels exhibited a low coefficient of friction (COF) value (0.06) in PBS, and the incorporation of polyphenol‐armored nanoparticles did not increase the COF.

### 2.7 SME‐Response Release and In Vivo Retention of the Hydrogel

The nMSN‐(PFe/CSWY)_3_/DHA hydrogel enabled the dynamic release of nanoparticles in response to the acidic SME, which was attributed to the pH‐sensitive imine bonds between the amino groups on the nanoparticles and aldehyde groups on the OHA‐DA chains. To stimulate in vivo release behavior, hydrogels prepared with Cy5.5‐labeled nMSN‐(PFe/CSWY)_3_ nanoparticles were immersed in pH 6.5 PBS supplemented with hyaluronidase. Hydrogels incubated in PBS (pH 7.4) containing hyaluronidase served as controls. As shown in Figure S11, the release of the Cy5.5‐MSN‐(PFe/CSWY)_3_ nanoparticles was significantly accelerated at pH 6.5, with 17.8% ± 0.4% of the nanoparticles released within 168 h. By contrast, only 14.7% ± 0.7% of the nanoparticles were released at pH 7.4. The nMSN@140‐(PFe/CSWY)_3_/DHA hydrogel demonstrated biocompatibility after immersing it into PBS (pH 6.5) containing hyaluronidase. It retained 90.5% ± 2.4%, 71.9% ± 6.4%, 59.2% ± 3.0%, and 20.5% ± 5.8% of its initial weight after 3, 7, 10, and 14 days of incubation, respectively (Figure S12).

To verify that the hydrogel can prolong the retention of polyphenol‐armored nanoparticles in a harsh joint cavity, Cy5.5‐labeled nanoparticles, with or without hydrogel encapsulation, were injected into the joint cavities of rats and subsequently tracked using a live imaging system (Figure 3J). The results indicated that the fluorescent signals of the Cy5.5‐MSN‐(PFe/CSWY)_3_ nanoparticle groups gradually decreased over time and completely disappeared after 14 days of post‐injection. By contrast, fluorescent signals of the Cy5.5‐MSN‐(PFe/CSWY)_3_/DHA hydrogel group were evident on Day 14, revealing the prolonged retention of nanoparticles after hydrogel encapsulation.

### 2.8 In Vitro Cytocompatibility and Gene Transfection

**FIGURE 4:**
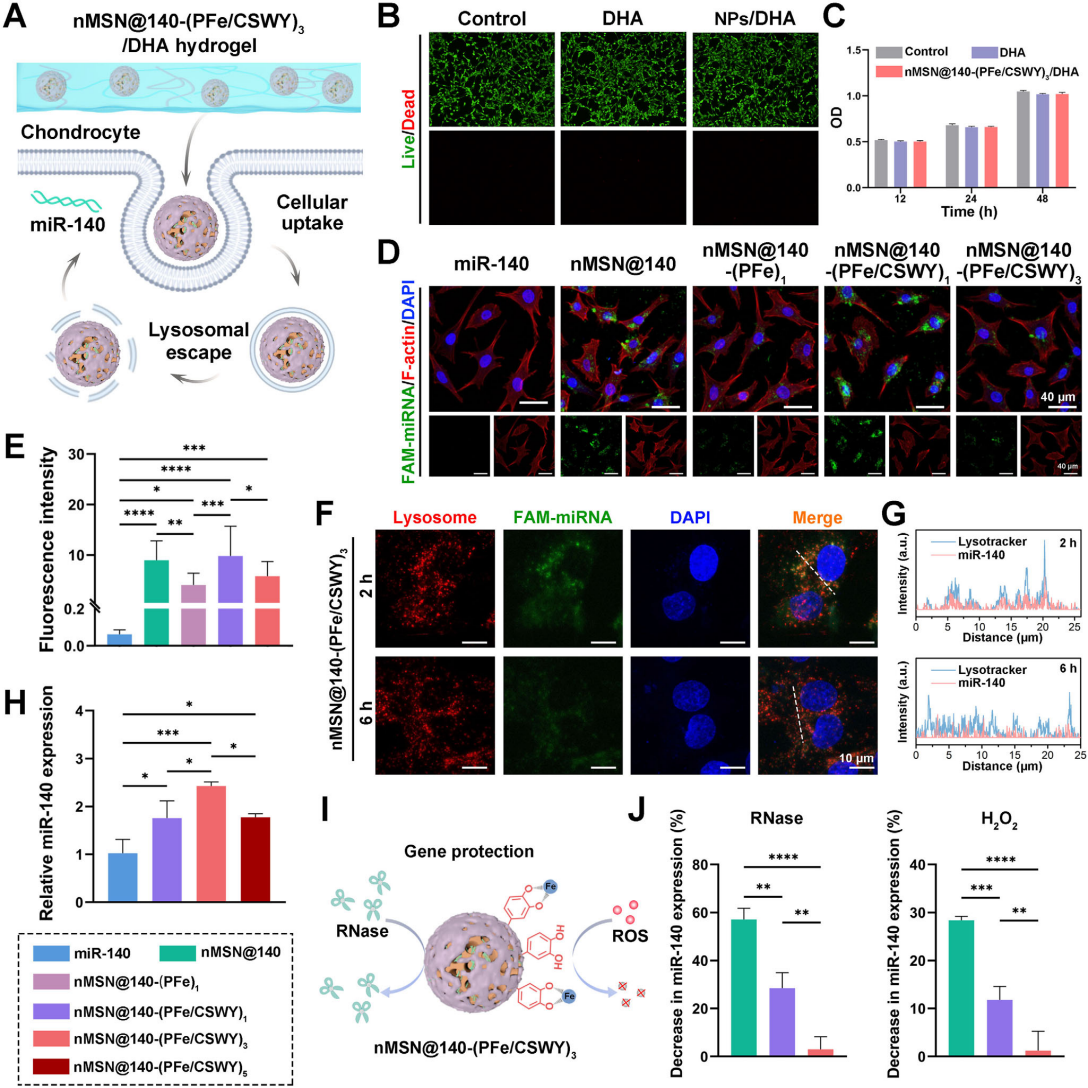
Gene protection and transfection. (A) Illustration of the cellular uptake and lysosomal escape of the nMSN@140‐(PFe/CSWY)_3_ nanoparticles released from the hydrogel. (B) Live/dead staining images and (C) MTT assay of the chondrocytes after coculture with hydrogels. (D) CLSM images and (E) fluorescence intensity of the chondrocytes after 6 h of incubation with the nanoparticles. (F) CLSM images and (G) colocalization of the nanoparticles and lysosomes in chondrocytes after 2 or 6 h of incubation. (H) Relative miR‐140 expression level in chondrocytes with various treatments. (I) Illustration of the nanoparticles physically shielding RNase and chemically eliminating ROS for gene protection. (J) Percentage decrease in miR‐140 expression levels for various nanoparticles following pre‐treatment with RNase or H_2_O_2_. All data are represented as mean ± SD (*n* ≥ 3). ^*^ *p* < 0.05, ^**^ *p* < 0.01, ^***^ *p* < 0.001, and ^****^ *p* < 0.0001.

The nMSN@140‐(PFe/CSWY)_3_ nanoparticles released from the hydrogel can internalize into chondrocytes and escape from lysosomes for miR‐140 transfection (Figure 4A). The cytocompatibility of the nMSN@140‐(PFe/CSWY)_3_/DHA hydrogel was assessed by coculturing with chondrocytes. Live/dead staining images indicated that numerous live chondrocytes were observed in the nMSN@140‐(PFe/CSWY)_3_/DHA hydrogel group, with negligible numbers of dead cells (Figure 4B). The cell count kit (CCK)‐8 results show that the optical density (OD) of the nMSN@140‐(PFe/CSWY)_3_/DHA hydrogel group was comparable to that of the control group (Figure 4C), indicating the cytocompatibility of the hydrogel.

The uptake of FAM‐labeled miR‐140, with or without nanoparticle loading, was visualized using confocal laser scanning microscopy (CLSM) after 6 h of co‐culturing with chondrocytes. As shown in Figure 4D,E, and Figure S13, pure miR‐140 was hardly taken up by chondrocytes owing to the negatively charged cell membrane barrier. By contrast, at least 65 times, FAM fluorescent intensity was detected in all FAM‐miR‐140‐loaded nanoparticle groups based on electrostatic attraction. The uptake of the nMSN@140‐(PFe/CSWY)_3_ nanoparticles by chondrocytes was further evaluated using isolated neonatal rat femoral heads. According to the fluorescence images in Figure S14, the nanoparticles were uniformly distributed in the surface and deep layers of the femoral head and were located around the nucleus after 48 h of incubation. This indicates that the nMSN@140‐(PFe/CSWY)_3_ nanoparticles could penetrate the dense cartilage ECM and internalize into chondrocytes. The co‐localization of the nMSN@140‐(PFe/CSWY)_3_ nanoparticles and lysosomes in chondrocytes was further assessed using CLSM (Figure 4F,G). An overlap of green and red fluorescence signals, representing FAM‐miR‐140‐loaded nanoparticles and lysosomes, respectively, was observed after 2 h of incubation. However, these signals were partially separated after 6 h of incubation, suggesting that the nanoparticles had escaped from lysosomes owing to their positive charge‐mediated proton sponge effect [35].

The miR‐140 transfection efficiency was determined via real‐time quantitative polymerase chain reaction (qRT‐PCR) analysis. The chondrocytes treated with the various polyphenol‐armored nanoparticles exhibited at least a 1.8‐fold higher miR‐140 expression level compared to the free miR‐140 control group. Notably, while increasing the deposition from 1 to 3 cycles improved the transfection efficiency, further increasing the coating to five cycles resulted in a decrease in miR‐140 expression (Figure 4H). This confirms that three deposition cycles of the polyphenol armor represent the optimal formulation for maximizing miR‐140 transfection efficiency.

The polyphenol armor helps the nanoparticles maintain the activity of miR‐140 by shielding RNase and eliminating ROS in the SME, contributing to a higher gene transfection efficiency (Figure 4I). To verify this, miR‐140‐loaded nanoparticles with or without polyphenol armor were pretreated with RNase or H_2_O_2_ and subsequently co‐incubated with chondrocytes (Figure 4J). Significant reduction in transfection efficiency was observed in the nMSN@140 (57.1% ± 4.7% reduction with RNase, 28.4% ± 0.9% reduction with H_2_O_2_) and nMSN@140‐(PFe/CSWY)_1_ nanoparticle groups (28.5% ± 6.5% reduction with RNase, 11.8% ± 2.8% reduction with H_2_O_2_). However, the miR‐140 expression level of the nMSN@140‐(PFe/CSWY)_3_ nanoparticle group was negligibly affected after pretreatments (3.0% ± 5.2% reduction with RNase, 1.2% ± 4.0% reduction with H_2_O_2_). This reveals the gene‐protective effect of the polyphenol‐armored nanoparticles.

### 2.9 Alleviation of Mitochondrial Dysfunction

**FIGURE 5:**
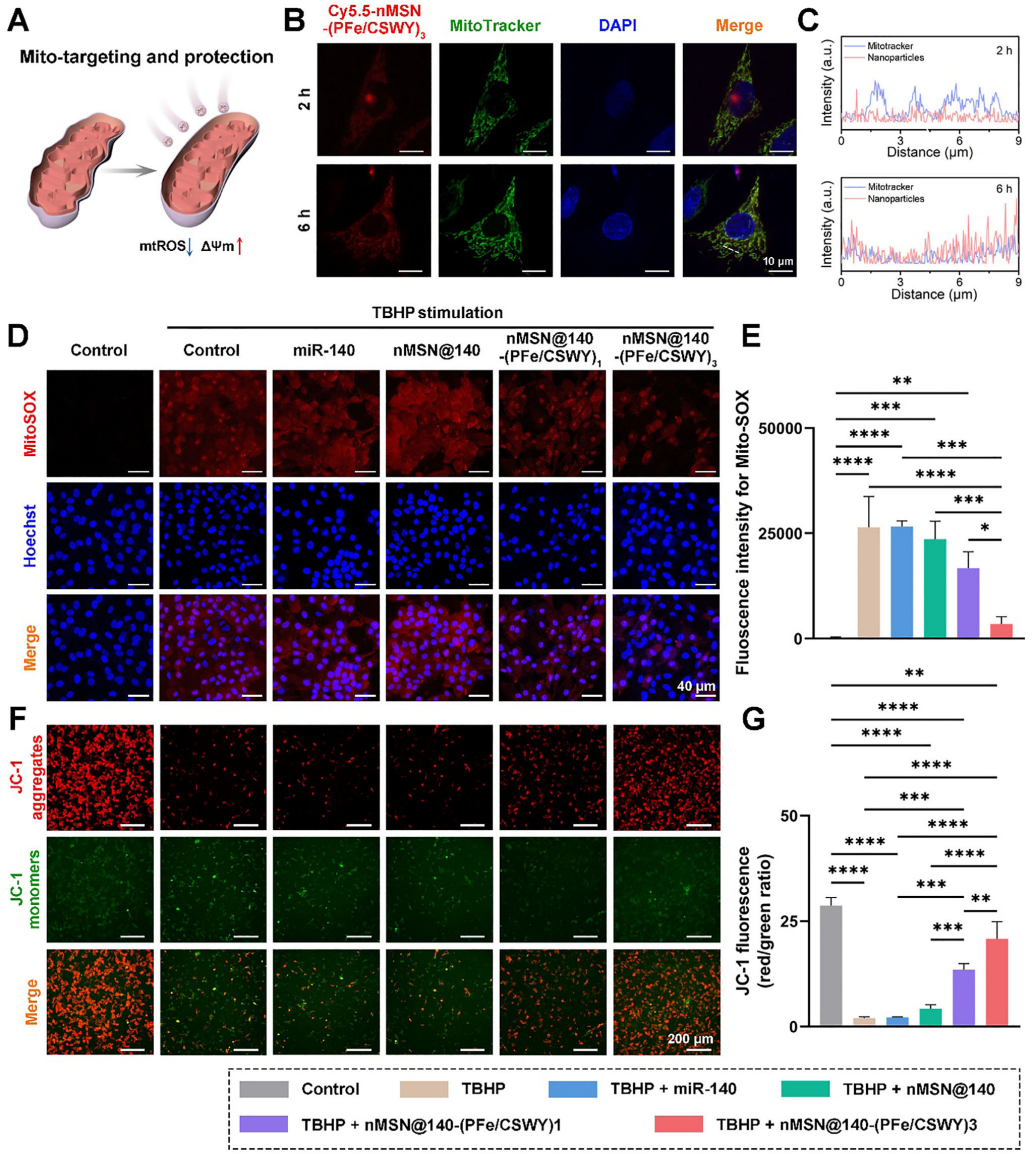
Alleviation of mitochondria dysfunction. (A) Illustration of the mitochondrial targeting and protection of the nMSN@140‐(PFe/CSWY)_3_ nanoparticles. (B) CLSM images and (C) colocalization of the nanoparticles and mitochondria in chondrocytes after 2 or 6 h of incubation. (D) MitoSOX fluorescence staining images and (E) corresponding quantitative analysis in TBHP‐treated chondrocytes after treatments with nanoparticles. (F) JC‐1 fluorescence staining images and (G) corresponding quantitative analysis in TBHP‐treated chondrocytes after treatments with nanoparticles. All data are represented as mean ± SD (*n* = 3). ^*^ *p* < 0.05, ^**^ *p* < 0.01, ^***^ *p* < 0.001, and ^****^ *p* < 0.0001.

Mitochondrial dysfunction is a hallmark of senescent chondrocytes and leads to excessive mtROS production, thereby creating a feedback loop that accelerates senescence [36]. The polyphenol‐armored nanoparticles can target electron‐rich mitochondria through electrostatic interaction and eliminate excess mtROS to restore mitochondrial dysfunction (Figure 5A). The mitochondria‐targeting capacity of nMSN‐(PFe/CSWY)_3_ nanoparticles was evaluated using a MitoTracker kit. The nanoparticles were labeled with Cy5.5 for tracking. As shown in Figure 5B, the nanoparticles were randomly distributed in chondrocytes after 2 h of incubation but gathered around the mitochondria after 6 h. Further analysis confirmed the co‐localization of the nanoparticles and mitochondria at 6 h, suggesting the mitochondria‐targeting capacity of the nanoparticles (Figure 5C). The mtROS levels in chondrocytes were evaluated after staining with MitoSOX. Tert‐butyl hydroperoxide (TBHP), a stable exogenous oxidative stress inducer that effectively penetrates cell membranes to generate radicals, was used to induce mitochondrial membrane potential depolarization and cellular senescence [37]. As shown in Figure 5D,E, TBHP stimulation increased the fluorescence signals in chondrocytes, and the signals were hardly attenuated after the treatment with miR‐140, suggesting negligible effects of miR‐140 on mtROS. By contrast, the nMSN‐(PFe/CSWY)_3_ nanoparticle group visibly reduced the mtROS level by 86.9% ± 6.6%, which is attributed to the antioxidative enzyme‐like activities of polyphenol armor. Furthermore, the nMSN‐(PFe/CSWY)_3_ nanoparticles also showed general intracellular ROS scavenging capability, as confirmed using a 2',7'‐dichlorodihydrofluorescein diacetate (DCFH‐DA) probe (Figure S15).

Mitochondrial membrane potential is associated closely with mitochondrial function [38]. Thus, the mitochondrial membrane potential in TBHP‐stimulated chondrocytes was assessed after staining with JC‐1 (Figure 5F). Upon TBHP stimulation, a collapse in membrane potential was observed in chondrocytes, as indicated by the weakened red fluorescence signals. By contrast, treatment with nMSN‐(PFe/CSWY)_3_ nanoparticles restored the membrane potential, similar to that of the negative control group. Quantitative analysis confirmed the superior effects of the nMSN‐(PFe/CSWY)_3_ nanoparticles in restoring the membrane potential, as indicated by the higher JC‐1 red/green ratio (20.8% ± 4.1%) compared with the miR‐140 (2.2% ± 0.1%), nMSN@140 (4.2% ± 1.0%), and nMSN@140‐(PFe/CSWY)_1_ (13.5% ± 1.4%) groups (Figure 5G).

### 2.10 Anti‐Senescence In Vitro

**FIGURE 6:**
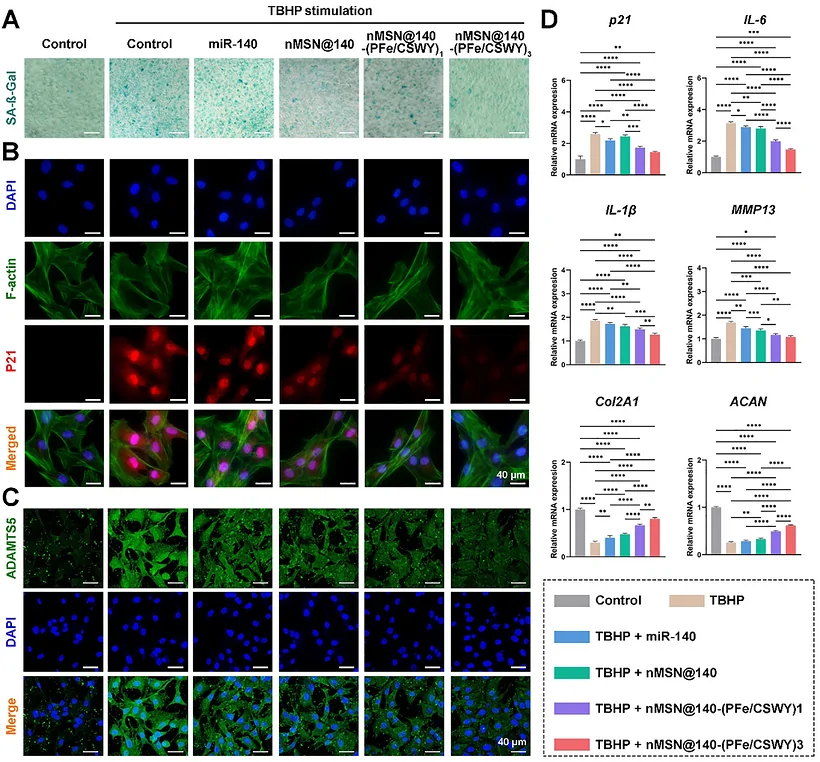
Anti‐senescence in vitro. (A) SA‐β‐Gal staining images and (B) immunofluorescence staining images of p21 and (C) ADAMTS5 in TBHP‐treated chondrocytes after treatments with various nanoparticles. (D) qRT‐PCR analysis results of the mRNA expression level in chondrocytes after treatments with various nanoparticles. All data are represented as mean ± SD (*n* = 3). ^*^ *p* < 0.05, ^**^ *p* < 0.01, ^***^ *p* < 0.001, and ^****^ *p* < 0.0001.

The nMSN@140‐(PFe/CSWY)_3_ nanoparticles with dual capabilities of miR‐140 delivery and targeted mtROS scavenging can mitigate chondrocyte senescence and suppress SASP factor production. The anti‐senescence efficacy was first evaluated using senescence‐associated β‐galactosidase (SA‐β‐Gal) staining (Figure 6A and Figure S16), a canonical senescence marker [39]. Fluorescence images revealed that TBHP stimulation significantly increased the average OD for SA‐β‐Gal from 1.5 ± 0.2 to 12.7 ± 0.5; however, it was attenuated to 3.3 ± 0.3 following treatment with nMSN‐(PFe/CSWY)_3_ nanoparticles. Subsequently, the expression levels of p21 and p16, which are core signaling molecules that trigger cell cycle arrest [40], were determined by immunofluorescence staining (Figure 6B, Figures S17 and S18). The results demonstrate that the nMSN‐(PFe/CSWY)_3_ group had remarkably lower fluorescence intensities for p21 and p16 than those of the TBHP (0.23‐fold for p21, 0.16‐fold for p16), miR‐140 (0.28‐fold for p21, 0.21‐fold for p16), nMSN@140 (0.38‐fold for p21, 0.26‐fold for p16), and nMSN‐(PFe/CSWY)_1_ (0.67‐fold for p21, 0.36‐fold for p16) groups.

The SASP factors [41], including the proinflammatory cytokines interleukin (IL)‐6 and tumor necrosis factor (TNF)‐α, and the protease a disintegrin‐like and metalloproteinase with thrombospondin type‐1 motifs (ADATMS)‐5, were assessed next. The results indicate decreased fluorescence signal levels for IL‐6, TNF‐α, and ADATMS‐5 in the nMSN‐(PFe/CSWY)_3_ nanoparticle group, compared with other treatment groups (Figure 6C, Figures S19 and S20). Moreover, treatment with nMSN‐(PFe/CSWY)_3_ nanoparticles led to upregulation of anabolic markers of collagen (Col)‐II (Figure S21). The qRT‐PCR analysis corroborated these findings at a genetic level, showing downregulation of senescence markers of p21 and SASP factors of IL‐1β, IL‐6, and matrix metallopeptidase (MMP)‐13, alongside up‐regulation of anabolic markers of Col2A1 and aggrecan (ACAN) in chondrocytes treated with the nMSN‐(PFe/CSWY)_3_ nanoparticles (Figure 6D). These results demonstrate that the nMSN@140‐(PFe/CSWY)_3_ nanoparticles attenuated chondrocyte senescence and remodeled the metabolic imbalance.

### 2.11 Hydrogel for Aged OA Therapy

**FIGURE 7:**
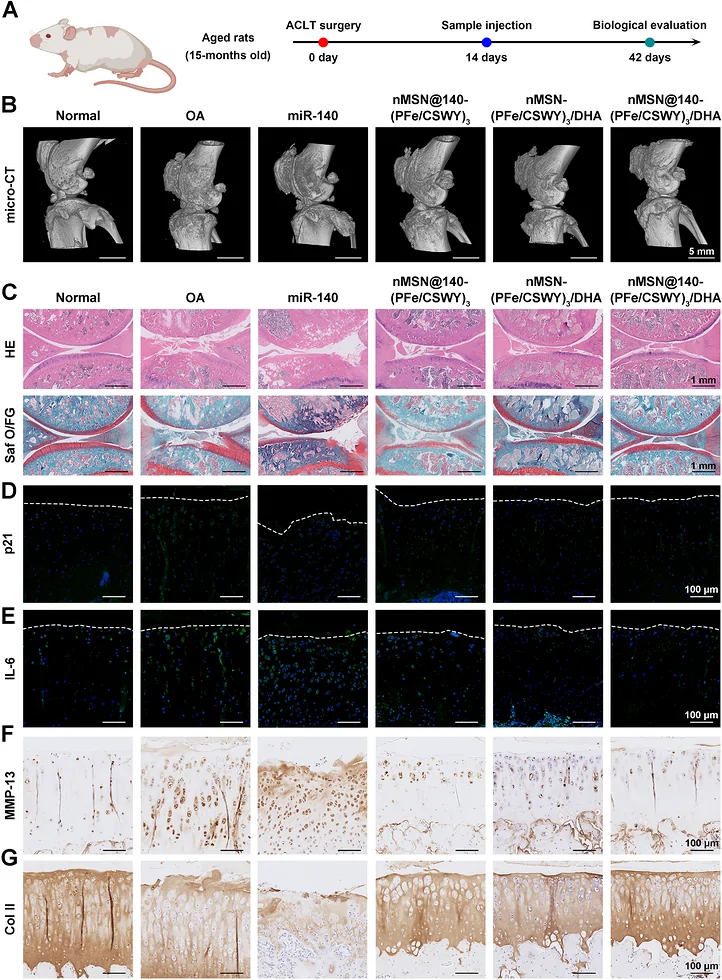
In vivo therapeutic efficiency of the nMSN‐(PFe/CSWY)_3_/DHA hydrogel in an aged OA rat model. (A) Experimental outline of the ACLT surgery‐induced OA rat model and following treatments. (B) Micro‐CT images of the rat joints 28 days after treatment. (C) HE and Saf O/fast green staining images of the rat joints. Immunofluorescent staining for (D) p21 and (E) IL‐6 of the rat joints. Immunochemical staining for (F) MMP13 and (G) Col II.

The in vivo therapeutic effect of the MSN@140‐(PFe/CSWY)_3_/DHA hydrogel was evaluated in an anterior cruciate ligament transection (ACLT)‐induced OA model in 15‐month‐old aged rats [42]. Fourteen days after ACLT surgery, the aged OA rats were randomly assigned into five groups receiving intra‐articular injections of PBS (OA group), miR‐140, MSN@140‐(PFe/CSWY)_3_ nanoparticles, MSN‐(PFe/CSWY)_3_/DHA hydrogels, or MSN@140‐(PFe/CSWY)_3_/DHA hydrogels. The aged rats without ACLT surgery (normal group) were set as the control. Twenty‐eight days after treatment, the rats were sacrificed to harvest knee joints for subsequent evaluation (Figure 7A).

The morphologies of rat joints 28 days after treatment were visualized using micro‐CT scanning (Figure 7B). Compared with the normal group, the OA group exhibited prominent osteophytes with a rough morphology on the articular surface. The treatment with miR‐140 failed to reduce these osteophytes. However, the MSN@140‐(PFe/CSWY)_3_/DHA hydrogel markedly suppressed osteophyte formation, resulting in a smooth articular surface. The quantitative results revealed that, compared to the OA group, the nMSN@140‐(PFe/CSWY)_3_/DHA hydrogel treatment group demonstrated significant improvements, including a 0.8‐fold increase in bone volume fraction (BV/TV) and a 1.1‐fold increase in trabecular thickness (Tb.Th) (Figure S22). These results indicate that the nMSN@140‐(PFe/CSWY)_3_/DHA hydrogel protected the subchondral bone from OA pathology‐induced degradation.

The histological changes of the articular cartilage were assessed using hematoxylin‐eosin (HE) and safranin O‐fast green staining (Figure 7C). Severe exfoliation of cartilage tissues and subchondral bone loss were observed in the PBS group. However, treatment with MSN@140‐(PFe/CSWY)_3_/DHA hydrogel significantly reduced the degenerative changes of joint tissues, contributing to an intact structural organization and a smooth cartilage surface, which was comparable to that of the normal group. The in vivo antisenescence effect was determined following immunofluorescence staining of p21 (Figure 7D and Figure S23A). The OA group exhibited a higher p21‐positive area (5.8% ± 0.8%), demonstrating senescence spreading progressively to surrounding cells through a typical bystander effect. The treatment with MSN@140‐(PFe/CSWY)_3_/DHA hydrogel alleviated the p21‐positive area to 1.7% ± 0.4%, which was superior to those of the miR‐140 (4.3% ± 0.5%), MSN@140‐(PFe/CSWY)_3_ nanoparticles (3.0% ± 0.7%), and nMSN‐(PFe/CSWY)_3_/DHA hydrogel (2.4% ± 0.4%) groups. The MSN@140‐(PFe/CSWY)_3_/DHA hydrogel also led to a sharp decrease in the positive area of SASP, including IL‐6 (76.1% ± 3.5% reduction) (Figure 7E and Figure S23B) and MMP‐13 (72.5% ± 8.9% reduction) (Figure 7F and Figure S23C), compared with the OA group. Moreover, immunohistochemistry staining analysis indicated a higher Col‐II expression level in the nMSN‐(PFe/CSWY)_3_/DHA hydrogel group (1.9‐fold compared with the OA group) (Figure 7G and Figure S23D). These results suggest that the MSN@140‐(PFe/CSWY)_3_/DHA hydrogel alleviated cartilage senescence and promoted anabolism in aged OA rats.

### 2.12 Underlying Mechanism of the Hydrogel

**FIGURE 8:**
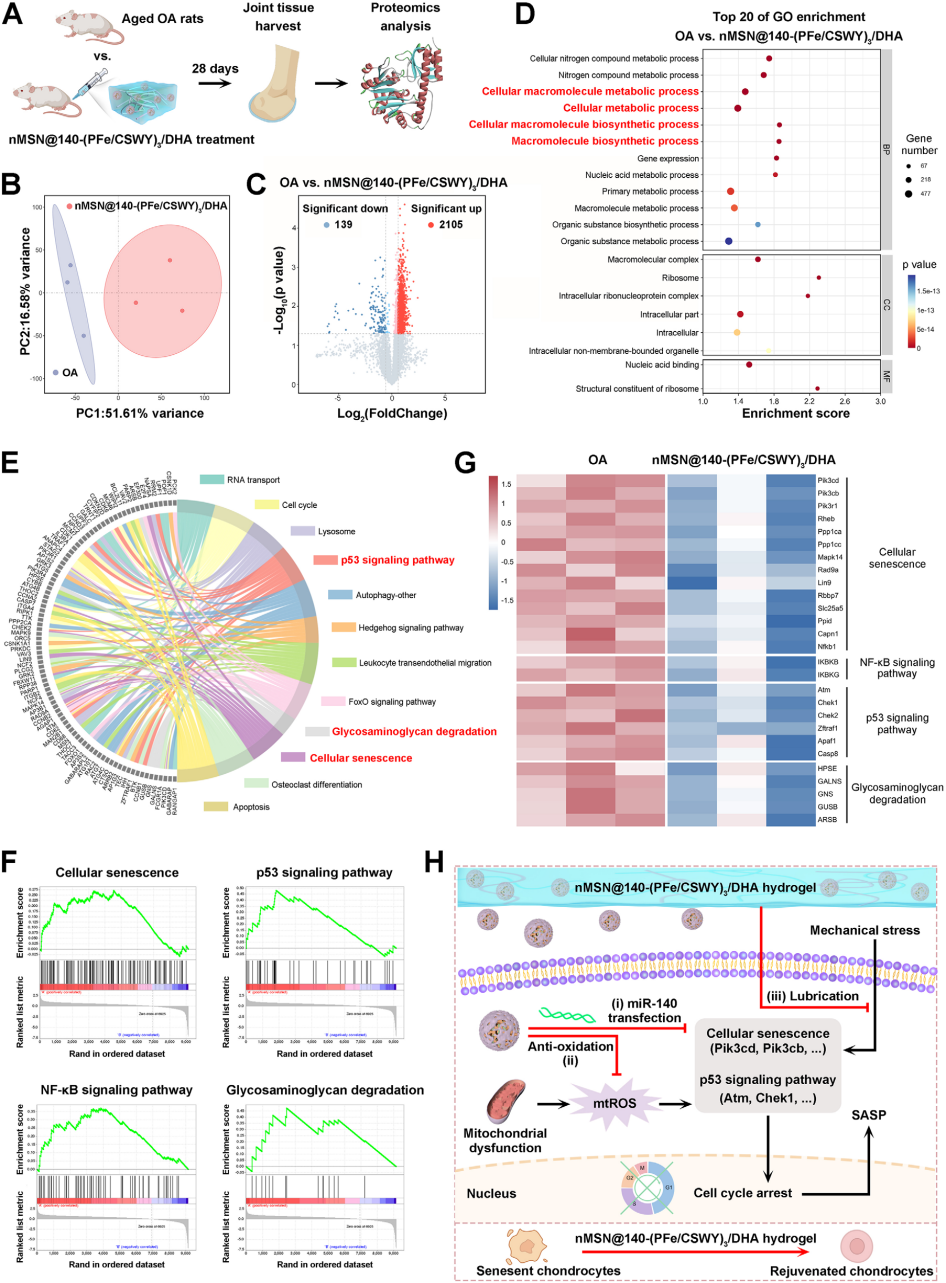
Proteomics data of aged OA rats and nMSN‐(PFe/CSWY)_3_/DHA hydrogel‐treated aged OA rats. (A) Experimental outline of the proteomics. (B) PCA of proteomics data. (C) Volcano plots illustrating proteins with biased patterns of expression between OA and nMSN‐(PFe/CSWY)_3_/DHA hydrogel groups. (D) GO enrichment analysis of the differentially expressed proteins. (E) Chordal plots of KEGG enrichment analysis of the differentially expressed proteins. (F) GSEA enrichment analysis. (G) Heat map indicating the differentially expressed proteins enriched in cellular senescence, NF‐κB signaling pathway, p53 signaling pathway, and glycosaminoglycan degradation. (H) Underlying therapeutic mechanism of the nMSN‐(PFe/CSWY)_3_/DHA hydrogel in treating aged OA rats.

To reveal the underlying therapeutic mechanism, the joint tissues of the aged OA rats, with or without nMSN@140‐(PFe/CSWY)_3_/DHA hydrogel treatment, were collected for proteomics analysis (Figure 8A). Principal component analysis (PCA) indicated a distinct separation between the OA and nMSN@140‐(PFe/CSWY)_3_/DHA treatment groups (Figure 8B), suggesting a significant difference in protein expression. In total, 2244 differentially expressed proteins (DEPs) were screened according to the selection criteria of |fold change| ≥ 1.5 and *p*‐value < 0.05, of which 2105 DEPs were upregulated, and 139 DEPs were downregulated (OA versus nMSN@140‐(PFe/CSWY)_3_/DHA) (Figure 8C).

Gene ontology (GO) enrichment demonstrated that the nMSN@140‐(PFe/CSWY)_3_/DHA hydrogel primarily modulated the DEPs associated with the metabolic processes, including the “cellular macromolecule metabolic process,” “cellular metabolic process,” “cellular macromolecule biosynthetic process,” and “macromolecule biosynthetic process” (Figure 8D and Figure S24). Kyoto Encyclopedia of Genes and Genomes (KEGG) analysis indicated the enrichment of DEPs in the “p53 signaling pathway” and “cellular senescence,” typical pathways to regulate cell cycles. In addition, the DEPs were also enriched in “glycosaminoglycan degradation,” a metabolism‐related signaling pathway (Figure 8E and Figure S25). Gene set enrichment analysis (GSEA) further confirmed that the nMSN@140‐(PFe/CSWY)_3_/DHA group exhibited a negative correction with “p53 signaling pathway,” “cellular senescence,” “NF‐κB signaling pathway,” and “glycosaminoglycan degradation” (Figure 8F, and Figure S26). Furthermore, the hydrogel significantly downregulated the DEPs enriched in these pathways (Figure 8G and Figure S27), suggesting an inhibitory effect of the hydrogel on cellular senescence. Based on the abovementioned results, the therapeutic mechanism of the nMSN@140‐(PFe/CSWY)_3_/DHA hydrogel in treating OA can be summarized as follows (Figure 8H). First, hydrogel‐mediated miR‐140 delivery directly downregulates the cellular senescence and p53 signaling pathways for antisenescence effects. Second, the hydrogel exhibits targeted elimination of mtROS, mitigating mitochondrial dysfunction and reducing the ROS‐triggered cellular senescence process. Third, the hydrogel adheres to cartilage tissue and provides reliable lubrication, reducing joint friction and, thus, mitigating the cellular senescence process caused by mechanical stress.

## 3 Discussion

Aged OA is characterized by severe chondrocyte senescence, driven by the combined effects of aging and cellular stress. Therapeutic strategies to mitigate aging‐associated pathologies include senolytics (inducing senescent cell death) [43], and senomorphics (inhibiting senescence‐related pathways) [3]. Although senolytics can damage healthy cells, senomorphic therapies offer superior safety. However, their efficacy in treating aged OA is significantly hampered by their low bioavailability owing to the ROS‐rich SME, the vascular/lymphatic clearance system in the joint cavity, and multiple biological barriers posed by the cartilage ECM, cell membrane, and lysosomes. To address these limitations, an injectable, adhesive, and lubricating hydrogel depot was developed that incorporates polyphenol‐armored nanoparticles as a platform for senomorphic miR‐140 gene delivery. This platform offers several advantages. First, the catechol groups in the hydrogel enhance anchoring on the cartilage surface, creating a localized depot for polyphenol‐armored nanogenes that extends intra‐articular retention to at least 14 days. This contrasts sharply with unencapsulated nanogenes, which are completely cleared within the same period. The hydrogel‐mediated prolonged therapeutic duration minimizes the risks of infection caused by frequent injections. Second, the polyphenol armor, with optimized deposition cycles and antioxidative enzyme‐like catechol/quinone‐iron redox pairs, acts as a protective barrier. It prevents the premature leakage of preloaded miR‐140 from nanoparticles while preserving miR‐140 activity by shielding it from RNase and eliminating ROS. This polyphenol armor‐mediated protection overcomes the inherent susceptibility of miR‐140 to enzymatic degradation and oxidative damage in the SME. Third, the CSWY peptide segments and positively charged surface of the polyphenol armor enable nanogenes to target the cartilage ECM, traverse cell membranes, and escape from lysosomes, significantly promoting the efficacy of miR‐140 transfection. Collectively, the hydrogel‐based platform with polyphenol‐armored nanoparticles achieved efficient and sustained miR‐140 transfection, downregulating cellular senescence and p53 pathways in chondrocytes.

Aging further contributes to mitochondrial dysfunction and ECM degeneration, causing excessive mtROS accumulation and increased joint friction. These processes establish a vicious cycle in which friction‐induced mechanical stress and mtROS‐induced oxidative stress act as persistent drivers of chondrocyte senescence. Previous efforts have been devoted to the fabrication of hydrogels that mimic synovial fluid lubrication or exert antioxidative effects [18, 44]. However, such hydrogels commonly lack sufficient bioadhesiveness, compromising their ability to maintain stable anchorage on cartilage surfaces for sustained lubrication. Furthermore, most antioxidative hydrogels show limited efficacy in targeted mtROS scavenging. In this study, the hydrogel possesses abundant adhesive catechol groups and lubricating carboxyl groups, enabling in situ anchorage onto cartilage tissues to reduce cartilage friction reliably. This combination of adhesion and lubrication reduces hydrogel dislodgement, preserving its extended lubricating function. Moreover, the polyphenol armor, composed of a positively charged CSWY layer and an antioxidative enzyme‐like PFe layer, targets electron‐rich mitochondria and subsequently eliminates mtROS. In short, the hydrogel provides localized lubrication and targeted mtROS scavenging to reduce the driving factors of aging.

## 4 Conclusions

An injectable, adhesive, and lubricating hydrogel depot with polyphenol‐armored nanogenes was constructed for suppressing chondrocyte senescence in aged OA. This material design offers several unique advantages. First, the hydrogel possesses abundant adhesive catechol groups and lubricating carboxyl groups, enabling in situ anchorage onto cartilage tissues to reliably reduce cartilage friction. This combination of adhesion and lubrication of hydrogel minimizes the fall off of hydrogels from cartilage, thereby preserving its extended lubricating function. Second, the hydrogel serves as a local depot for the polyphenol‐armored nanogenes, prolonging their intra‐articular retention to at least 14 days and thus minimizing the risk of infection caused by frequent injection. Third, the polyphenol armor with optimized deposition cycles acts as a protective barrier, preventing the premature leakage of preloaded miR‐140 from the nanoparticles, while preserving its activity against RNase and ROS enriched in SME. Furthermore, the positively charged CSWY in polyphenol armor overcomes the biological barriers of cartilage ECM, cell membranes, and lysosomes, significantly promoting miR‐140 transfection efficacy. Fourth, the anti‐oxidative enzyme‐like PFe layer in polyphenol armor restores mitochondrial dysfunction via targeted mtROS scavenging. By combining advanced gene delivery, sustained lubrication, and targeted mtROS scavenging capabilities, this hydrogel significantly mitigates chondrocyte senescence and SASP production in aged OA rats, indicating its clinical potential.

## 5 Experimental Section

### 5.1 Materials

Dopamine‐HCl (DA) was purchased from Sigma–Aldrich, USA. Hyaluronic acid (HA; Mn = 20 000–40 000), chitosan (CS; viscosity = 100–200 mPa·s), adipic acid dihydrazide (ADH), 1‐hydroxybenzotriazole (HOBT), iron chloride hexahydrate, and sodium periodate were purchased from MACKLIN, China. 4‐Morpholineethanesulfonic acid (MES), N‐hydroxysuccinimide (NHS), and 1‐ethyl‐3‐(3‐dimethylaminopropyl)‐carbodiimide hydrochloride (EDC) were purchased from RHAWN, China. WYRGRL peptide was purchased from Shanghai Apeptide Co., Ltd, China. nMSN nanoparticles were purchased from Jiangsu Zhichuan Technology Co., Ltd, China. miR‐140‐5p and FAM‐miR‐140‐5p were purchased from HANBIO, China.

### 5.2 Synthesis of the nMSN@140‐(PFe/CSWY)_3_ Nanoparticle

The nMSN@140‐(PFe/CSWY)_3_ nanoparticles were synthesized via the following three steps. The first step involves miR‐140 loading. Briefly, 40 µg of miR‐140 powders was added into 10 mL of the nMSN nanoparticle suspension (1 mg/mL; RNase‐free water as the solvent), and allowed ultrasonic treatment for 30 min. The nMSN@140 nanoparticles were collected from the resultant suspension by centrifugation (10 000 rmp, 5 min) and washed with RNase‐free water for two times. The second step involves the deposition of the PFe layer. Briefly, the obtained nMSN@140 nanoparticles were re‐dispersed in 10 mL of RNase‐free water and mixed with 10 mL of a mixture containing 5 mg of DA and 0.8 mg of FeCl_3_·6H_2_O. Subsequently, 20 µL of NaOH solution (1 g/mL) was introduced into the suspension to enable polymerization of DA under an alkaline environment. After 20 min of reaction, the nMSN@140‐(PFe)_1_ nanoparticles were collected by centrifugation (10 000 rmp, 5 min) and washed with RNase‐free water for two times. The third step involves the deposition of the CSWY layer. Briefly, the obtained nMSN@140 nanoparticles were re‐dispersed in 10 mL of RNase‐free water and mixed with 10 mL of CSWY solution (0.5 mg/mL). After 10 min of reaction, the nMSN@140‐(PFe/CSWY)_1_ nanoparticles were collected by centrifugation (10 000 rmp, 5 min) and washed with RNase‐free water for two times. By repeating the step two and step three for three times, the nMSN@140‐(PFe/CSWY)_3_ nanoparticles were synthesized. The Cy5.5‐nMSN‐(PFe/CSWY)_3_ nanoparticles were prepared by grafting Cy5.5‐NHS (APExBIO, USA) onto the nMSN nanoparticles, followed by repeated deposition of PFe and CSWY layer for three times.

### 5.3 Characterization of CSWY

For the NMR characterization, freeze‐dried powders of CS, CSWY, and the WYRGRL peptide were dissolved in D_2_O. The spectra were recorded using a Bruker AV II‐600 MHz spectrometer (Switzerland). For the BCA assay, a standard curve was established using a series of WYRGRL peptide solutions (0, 25, 50, 100, 200, 300, and 400 µg/mL) following the standard protocol. Subsequently, a 1 mg/mL CSWY solution was tested to determine the peptide content.

### 5.4 Characterizations of Nanoparticles

The morphology of the nanoparticles was observed by transmission electron microscopy (TEM; JEM‐F200, JEOL, Japan) and scanning electron microscopy (SEM; Sigma 360, ZEISS, Germany). The zeta potential of the nanoparticles in each synthesis process was determined using a Zeta potential analyzer (Zetasizer Nano ZS90, Malvern, UK) at 25°C. The chemical states of the nanoparticles were investigated by XPS (Nexsa, Thermo Fisher, USA) with a monochromatic Al Kα X‐ray excitation source (hν = 1486.6 eV). The chelation between PDA and iron ions in polyphenol armor was evaluated via UV–vis spectroscopy. Briefly, 10 mg of dopamine and 10 mg of iron chloride hexahydrate were dissolved in 10 mL of deionized water. Then 10 µL of NaOH solution (100 mg/mL) was added to the mixture. The solution was stirred for 30 min to obtain PFe, followed by a 200‐fold dilution 200 for UV–vis analysis (TU‐1901, Puxi, China) in the wavelength range of 250–800 nm. The samples prepared without the addition of iron chloride hexahydrate (PDA), and samples prepared without dopamine and NaOH (Fe) were used for comparison. The encapsulation efficiency of the nanoparticles for FAM‐miR‐140 was determined by measuring the fluorescence intensity of the supernatant with a fluorescence microplate reader (SynergyH1, BioTek, USA) after centrifugation.

### 5.5 Release of FAM‐miR‐140 from Nanoparticles

The release of FAM‐miR‐140 from nanoparticles was determined using a fluorescence microplate reader (SynergyH1, BioTek). Briefly, 5 mL of nanoparticle suspension (1 mg/mL) was added to the dialysis bag (14000 DA) and then immersed in PBS at 6.5. At a certain time interval, the saline was collected for fluorescence intensity detection.

### 5.6 Antioxidative Enzyme‐Like Activity

The general antioxidative activity of nanoparticles was determined by the DPPH assay. Briefly, 1 mL of the nanoparticle suspension (1 mg/mL) was introduced into 3 mL of DPPH (MACKLIN) solution (40 µg/mL, methanol as the solvent). After 3 h of reaction, the absorption of the mixture at 516 nm was measured using a UV–vis spectrophotometer (TU‐1901, Puxi, China). The SOD‐like activity of nanoparticles was evaluated according to the standard procedures with an SOD activity assay kit (Solarbio, China). The CAT‐like activity of the nanoparticles was measured using an oxygen dissolution tester (Mettler Toledo, China). Briefly, 200 µL of nanoparticles (1 mg/mL) was introduced into 10 mL of deionized water containing 200 µL of H_2_O_2_ (30%). At specific time intervals (0, 3, 6, 9, 12, 15 min), the oxygen concentration of the suspension was measured. Oxygen generation was calculated by subtracting the initial oxygen concentration from the measured value. The PFe sample was prepared by dissolving 10 mg of dopamine and 10 mg of iron chloride hexahydrate in 10 mL of deionized water, followed by the addition of 10 µL of NaOH solution (100 mg/mL) and stirring for 30 min. The PDA sample was prepared by adding 10 µL of NaOH (100 mg/mL) solution to 10 mL of dopamine solution (1 mg/mL) and stirring for 30 min. The nMSN, Fe, and CSWY samples were prepared at a concentration of 1 mg/mL.

### 5.7 Synthesis of the nMSN@140‐(PFe/CSWY)_3_/DHA Hydrogel

The nMSN@140‐(PFe/CSWY)_3_/DHA hydrogel was prepared by encapsulating the nMSN@140‐(PFe/CSWY)_3_ nanoparticles into a HA‐based polymer matrix. Briefly, 200 µL of the nMSN@140‐(PFe/CSWY)_3_ nanoparticle suspension was introduced into 600 µL of the HA‐ADH solution (2 wt.%) under stirring. Then the suspension was mixed with 100 µL of OHA‐DA solution (10 wt.%), and the hydrogel was formed within a few seconds.

### 5.8 Characterizations of HA Derivatives

Briefly, freeze‐dried powders of HA, OHA, OHA‐DA, and HA‐ADH were dissolved in D_2_O. The spectra were recorded using a Bruker AV II‐600 MHz spectrometer (Switzerland). The modification ratios were calculated by using the N‐acetylmethyl protons as an internal reference.

### 5.9 Characterizations of Hydrogels

The rheological behavior of the nMSN‐(PFe/CSWY)_3_/DHA hydrogel was investigated using a rheometer (MCR302, Anton Paar, Austria) at 37°C. To determine the gelation time of the hydrogel, the precursor was loaded into the gap between the plates, and time sweeps were performed at 1% strain amplitude and 1 Hz frequency amplitude. To confirm the dynamical crosslinking of the hydrogel, step‐stain sweeps were performed at 1% or 300% strain amplitude and 1 Hz frequency amplitude after loading the as‐prepared hydrogel into the plates. The microstructure of the hydrogels was observed using SEM (Sigma 360, ZEISS). Prior to observation, the hydrogel samples were freeze‐dried at ‐80°C for 48 h, cryo‐fractured in liquid nitrogen to expose their cross‐section, and sputter‐coated with gold. The compressive properties of the hydrogels were evaluated using a universal testing machine (UTM; Instron 5567, USA) equipped with a 100 N load cell. Cylindrical hydrogel samples (10 mm in diameter, 10 mm in height) were placed between two parallel plates and compressed at a loading rate of 1 mm/min until structural failure occurred. The compressive strength was calculated as the maximum load divided by the initial cross‐sectional area of the hydrogel. The toughness was determined by integrating the area under the stress–strain curve from the onset of loading up to the point of fracture. The adhesion strength of the hydrogels was quantified via a lap‐shear test using a dynamic mechanical analyzer (TA Instruments Q800, USA). Briefly, the hydrogel precursor solution was applied to the interface and sandwiched between two pieces of fresh porcine skin, creating a defined overlapping area. After 10 min incubation period to ensure complete in situ hydrogel formation, the sandwiched system was fixed to a pair of clamps and stretched at a loading rate of 5 mm/min until adhesion failure. The shear adhesion strength was calculated by dividing the maximum detachment force by the initial contact area. The interfacial toughness was determined using a 180° peeling test. Briefly, 500 µL of the hydrogel precursor was applied between two pieces of fresh porcine skin. After gelation, the unbonded ends of the porcine skin were fixed to a pair of clamps of a universal testing machine (Instron 5567, USA) and stretched at a loading rate of 5 mm/min. The interfacial toughness was calculated as two times the average steady‐state peeling force divided by the width of the hydrogel‐tissue interface. The COF of hydrogels in PBS (pH 7.4) was measured using a high‐speed friction and wear testing machine (MTF‐R4000, China) with a loading of 1 N and frequency of 1 Hz.

### 5.10 Release of Cy5.5‐nMSN‐(PFe/CSWY)_3_ Nanoparticles from Hydrogels

The release of the Cy5.5‐nMSN‐(PFe/CSWY)_3_ nanoparticles from the hydrogels was determined using a fluorescence microplate reader (SynergyH1, BioTek). Briefly, the as‐prepared Cy5.5‐nMSN‐(PFe/CSWY)_3_/DHA hydrogel samples (1 × 1 × 1 cm^3^) were introduced into 10 mL of PBS (pH 7.4 or 6.5) containing hyaluronidase (50 U/mL). After incubation at 37°C with continuous stirring for 1, 2, 3, 5, and 7 days, 1 mL of the release medium was collected for detection and immediately replaced with an equal volume of fresh medium.

### 5.11 Biodegradability of Hydrogels

The as‐prepared nMSN@140‐(PFe/CSWY)_3_/DHA hydrogels (1 × 1 × 1 cm^3^) were immersed in 5 mL of PBS (pH 6.5) containing 100 U/mL hyaluronidase. After incubation at 37°C for 3, 7, 10, and 14 days, the samples were freeze‐dried and weighed. The remaining mass was calculated as the ratio of the residual weight to the initial weight.

### 5.12 Chondrocyte Isolation and Cell Culture

Primary chondrocyte was isolated from the knee joints of 1‐week‐old Sprague Dawley rats. Briefly, following euthanasia, the rats were surface‐sterilized with 75% ethanol. Hyaline cartilage was then dissected from femoral and tibial surfaces, minced into approximately 1 mm^2^ pieces, and subjected to extracellular matrix (ECM) digestion using 2 mg/mL collagenase II (Sigma–Aldrich) at 37°C for 4 h. Harvested chondrocytes were cultured in DMEM/F12 medium (Gibco) supplemented with 10% fetal bovine serum (FBS; Corning) and 1% penicillin/streptomycin (P/S; Gibco) under standard conditions (37°C, 5% CO_2_). Chondrocytes at passages 3–4 (P3‐P4) were used for subsequent experiments.

### 5.13 Cytocompatibility of Hydrogels

The cytocompatibility of the hydrogels were evaluated after incubating with chondrocytes. The chondrocytes were seeded on a 24‐well plate. After 12 h, the hydrogel sample (Φ = 10 mm, h = 2 mm) was added into the medium and co‐cultured for 1 day. The live and dead chondrocytes were observed using a fluorescence microscope (Olympus BX53, Japan) after staining with a calcein‐AM/PI kit (Beyotime, China). The viability of chondrocytes was determined using a cell count kit (CCK)‐8 (Beyotime).

### 5.14 Cellular Uptake of Nanoparticles

The in vitro cellular uptake of the FAM‐miR‐140‐loaded nanoparticles was investigated by coculturing with chondrocytes. The chondrocytes were cocultured with nanoparticles (20 µg/mL) for 6 h. Afterward, the chondrocytes were stained with Tetramethylrhodamine B isothiocyanate labeled phalloidin (Sigma–Aldrich) and 4',6‐diamidino‐2‐phenylindole (DAPI; Beyotime), followed by observation using a CLSM (Carl Zeiss LSM880). A total of two 7‐day‐old Sprague–Dawley (SD) rats were used, providing four femoral heads for the ex vivo experiments. Briefly, the rats were euthanized and sterilized with 75% ethanol. The femurs were isolated under sterile conditions, and the femoral heads were carefully separated from the femoral shafts using sterile surgical scissors. The isolated femoral heads were subsequently cultured in DMEM/F12 medium supplemented with 10% fetal bovine serum (FBS) and 1% penicillin‐streptomycin at 37°C, with the addition of 1 mg/mL FAM‐miR‐140‐loaded nMSN‐(PFe/CSWY)_3_ nanoparticles. After 24 h of co‐incubation, the femoral heads were washed with PBS, cryosectioned, and observed using a fluorescence microscope (Leica, Germany).

### 5.15 Lysosomal Escape of Nanoparticles

The escape of the FAM‐miR‐140‐loaded nanoparticles from the lysosome in chondrocytes was visualized using a CLSM (Carl Zeiss LSM880). The chondrocytes were cocultured with the nanoparticles (20 µg/mL) for 2 and 6 h. Afterward, the chondrocytes were stained with LysosomeTracker Red (KeyGEN BioTECH, China) for observation.

### 5.16 Gene Transfection

Chondrocytes were seeded in a 48‐well plate at a density of 10^4^ cells per well. The cells were then incubated with the nanoparticles (10 µg/mL) for 24 h. The expression level of miR‐140 was quantified using qRT‐PCR on a QuantStudio3 System (Applied Biosystems, USA). The primer sequence used was GTCCTCCTCTCCTTCCTTCTCATGAGGAGGACCTACCA.

### 5.17 Gene Protection

The gene protection of the nMSN@140‐(PFe/CSWY)_3_ nanoparticles was evaluated by detecting the miR‐140 expression level after co‐culturing the chondrocytes with H_2_O_2_ or RNase‐pretreated nanoparticles. Briefly, the nanoparticles (1 mg/mL) were treated with RNase (100 µg/mL) or H_2_O_2_ (200 µm) for 12 h and collected by centrifugation (10 000 rmp, 5 min). Afterward, the nanoparticles (10 µg/mL) were co‐cultured with chondrocytes for 24 h. The miR‐140 expression level in chondrocytes was detected by qRT‐PCR with a QuantStudio3 System (Applied Biosystems).

### 5.18 Mitochondrial Targeting of Nanoparticles

The co‐localization of the mitochondria and nanoparticles in chondrocytes was visualized using a CLSM (Carl Zeiss LSM880). After 2 and 6 h of co‐culture with the Cy5.5‐nMSN‐(PFe/CSWY)_3_ nanoparticles (20 µg/mL), the chondrocytes were stained with MitoTracker Green (Beyotime) for observation.

### 5.19 mtROS Scavenging

The mtROS level in TBHP‐damaged chondrocytes, with or without nanoparticle treatment, was evaluated using a CLSM (Carl Zeiss LSM880). The chondrocytes were seeded on a 48‐well plate. After 12 h of culture, the nanoparticles (40 µg/mL) and TBHP (50 µm) were added to the medium and co‐cultured for 6 h. The chondrocytes were stained with MitoSOX (Beyotime) for observation using a fluorescence microscope (Olympus BX53, Japan). The general intracellular ROS was evaluated using a DCFH‐DA (Beyotime) probe.

### 5.20 Mitochondrial Membrane Potential

The mitochondrial membrane potential in TBHP‐damaged chondrocytes, with or without nanoparticle treatment, was evaluated using a fluorescent microscope (Leica). The chondrocytes were seeded on a 48‐well plate. After 12 h of culture, the nanoparticles (40 µg/mL) and TBHP (50 µm) were added to the medium and co‐cultured for 12 h. The chondrocytes were stained with JC‐1 (Beyotime) for observation.

### 5.21 Anti‐Senescence In Vitro

The anti‐senescence capacity of the nanoparticles was evaluated by SA‐β‐Gal staining, immunofluorescence staining, and qRT‐PCR analysis. The chondrocytes were stimulated with TBHP (100 µm) and simultaneously treated with the nanoparticles (40 µg/mL) for 24 h. For SA‐β‐Gal evaluation, the chondrocytes were fixed with paraformaldehyde (Kelong, China) for 20 min and stained using an SA‐β‐Gal kit (Beyotime) before observation. For immunofluorescence staining, the chondrocytes were fixed with paraformaldehyde (Kelong) for 20 min, permeabilized with 0.3% Triton X‐100 (Beyotime) for 10 min, blocked with 5% bull serum albumin (BSA; Beyotime) for 1 h, followed by staining with p21 antibody (ABclonal, China), p16 antibody (Abclonal), collagen II antibody (Affinity, USA), ADAMTS5 antibody (ABclonal), TNF‐α antibody (Affinity), or IL‐1β antibody (Affinity) overnight before observation. For qRT‐PCR analysis, the gene (p21, IL‐6, IL‐1β, MMP13, Col2A1, ACAN) expression level was detected using a QuantStudio3 System (Applied Biosystems). The primer sequences are listed in Table S2.

### 5.22 In Vivo Retention Time

The in vivo retention time of the Cy5.5‐nMSN‐(PFe/CSWY)_3_ nanoparticles, with or without hydrogel encapsulation, was investigated using a live animal imaging system (IVIS Spectrum, PerkinElmer, USA). Adult SD rats (8–10 weeks old, approximately 250 g) were anesthetized via isoflurane inhalation. Subsequently, 200 µL of the free Cy5.5‐nMSN‐(PFe/CSWY)_3_ nanoparticles or the Cy5.5‐nMSN‐(PFe/CSWY)_3_/DHA hydrogel precursor was injected into the knee joint cavity. After specific time points (12 h, 1, 3, 5, 7, and 14 d), the rats were anesthetized again with isoflurane for fluorescence imaging.

### 5.23 Establishment of Aged OA Model and Hydrogel Treatment

Aged male Sprague Dawley rats (15‐month‐old, 800–1000 g) were purchased from Chengdu Dossy Experimental Animals Co., LTD. All the animal experiments were approved by the Animal Ethics Committee of West China Hospital of Sichuan University (20240919003), following the Guide for the Care and Use of Laboratory Animals published by the Chinese National Academy of Sciences. The OA model was established using an ACLT surgery. Briefly, the rats were anesthetized via intraperitoneal injection of sodium pentobarbital, and both hind limbs were shaved and sterilized. A longitudinal skin incision (approximately 1 cm) was made medial to the patellar ligament, followed by blunt dissection to expose the joint capsule. The joint capsule was incised, and the patella was gently dislocated laterally while flexing the knee to approximately 90° to fully expose the anterior cruciate ligament (ACL). The ACL was then transected using micro‐scissors, and complete transection was confirmed by a positive anterior drawer test. Following irrigation of the joint cavity with sterile saline, the joint capsule, subcutaneous tissue, and skin were closed sequentially in layers. To prevent post‐surgical infection, the rats received intramuscular injections of penicillin for three consecutive days. The ACLT‐induced osteoarthritis models were confirmed to be successfully established fourteen days post‐surgery. Subsequently, the rats were anesthetized via intraperitoneal injection of sodium pentobarbital, and the samples (200 µL) were implanted into the joint cavity via intra‐articular injection.

### 5.24 Micro‐CT

After 28 days of post‐implantation, the rats were sacrificed, and the joint tissues were harvested. The microstructure of the rat joint was observed using a micro‐CT imaging system (Quantum GX3, PerkinElmer).

### 5.25 Histological Analysis

The joint tissues were fixed in paraformaldehyde for 12 h, decalcified in EDTA for 6 weeks, dehydrated through a graded ethanol series, embedded in paraffin, and sectioned at 5 µm. The sections were collected exactly at the level where the meniscus becomes discontinuous. For histomorphological evaluation, the sections were stained with HE and safranin O‐fast green and observed using a microscope (NI‐E, Nikon). For immunohistochemical staining, the sections were treated with 3% H_2_O_2_ for 10 min, blocked with 5% BSA for 1 h, and incubated overnight at 4°C with primary antibodies against MMP13 (PTG, USA) and Col II (Arigobio, China). After incubation with species‐matched secondary antibodies for 1 h, immunoreactivity was visualized using 3,3′‐diaminobenzidine (DAB; Boster, China), and nuclei were counterstained with hematoxylin (Beyotime). Sections were mounted in neutral resin for examination under the microscope (NI‐E, Nikon). For immunofluorescence staining, the sections were similarly treated with H_2_O_2_, blocked with BSA, and incubated with primary antibodies against IL‐6 (Boster) or p21 (ABclonal). Following incubation with fluorescent secondary antibodies for 1 h, the sections were mounted in Antifade Mounting Medium (Beyotime) containing DAPI (Beyotime) and observed using a CLSM (A1RMP+, Nikon).

### 5.26 Proteomics Analysis

At 28 days post‐treatment, joint tissues were harvested from a randomly selected subgroup (*n* = 3 per group) of rats in the OA and nMSN@140‐(PFe/CSWY)_3_/DHA hydrogel groups for proteomic analysis. The joint tissues were collected and subsequently submitted to Novogene Co., Ltd for proteomics analysis. Significantly differentially expressed proteins were identified with the criteria of |fold change| ≥1.5 and *p* value <0.05.

### 5.27 Statistical Analysis

The data were analyzed using OriginPro and Prism 9 software. The results are expressed as mean ± standard deviation. Statistical significance between different groups or treatments was assessed using one‐way analysis of variance (ANOVA) followed by Tukey's multiple comparisons test with a value of ^*^ *p* < 0.05, ^**^ *p* < 0.01, ^***^ *p* < 0.001, and ^****^ *p* < 0.0001.

## Author Contributions

L.Y., R.Y., C.X., W.F. designed experiments; L.Y., R.Y., C.X., W.F. developed the methodology; L.Y., R.Y., T.Z., T.X., Y.L., Y.H., M.G., L.Z. fabricated the materials and conducted the characterization; L.Y. drafted the paper; C.X. and W.F. supervised this work; All authors reviewed and edited the manuscript.

## Conflicts of Interest

The authors declare no conflicts of interest.

## Supporting information




**Supporting File**: advs75792‐sup‐0001‐SuppMat.docx.

## Data Availability

The data that support the findings of this study are available from the corresponding author upon reasonable request.
