# Supplementary material for: Bioadhesive Hydrogel With Polyphenol‐Armored Nanogene Rejuvenates Chondrocyte Senescence for Aged Osteoarthritis Therapy
Source: Adv Sci (Weinh). 2026 May 20;13(46):e00014. doi: 10.1002/advs.202600014 (PMC13336078; doi:10.1002/advs.202600014)
Supplement: Supplementary file 1 [file ADVS-13-e00014-s001.docx]

Supporting Information

Bioadhesive Hydrogel with Polyphenol-Armored Nanogene Rejuvenates Chondrocyte Senescence for Aged Osteoarthritis Therapy

*Liwei Yan, Runze Yang, Ting Zhou, Tianhao Xu, Yongqi Li, Yuelin Hu, Minghao Ge, Lei Zhang, Xiong Lu, Chaoming Xie***, Weili Fu**

**1. Methods**

**Synthesis of WYRGRL-modified CS (CSWY).** 0.16 g of chitosan (CS) powders were dispersed in 30 mL of 4-morpholineethanesulfonic acid (MES) buffer solution, followed by adding acetic acid dropwise until CS was completely dissolved. Then 10 mL of WYRGRL solution (8.5 mg/mL; MES buffer solution as the solvent), 1.07 g of 1-ethyl-3-(3-dimethylaminopropyl)-carbodiimide hydrochloride (EDC), and 0.39 g of n-hydroxysuccinimide (NHS) was sequentially introduced into the mixture. After 12 h of reaction under dark, the mixture was dialyzed against deionized (DI) water for 3 d and freeze-dried at -80°C to obtain CSWY powders.

**Synthesis of dopamine-modified oxidized hyaluronic acid (OHA-DA).** OHA-DA was synthesized via a two-step process. First, 1 g of hyaluronic acid (HA) powders were dissolved in 100 mL of DI water. Then 5 mL of NaIO_4_ solution (0.18 g/mL) was introduced dropwise into the HA solution. After 2 h of stirring under dark, 0.5 mL of glycol was added to terminate the oxidative reaction. The suspension was dialyzed against DI water for 3 d and freeze-dried at -80°C to obtain OHA powders. Second, 0.4 g of OHA powders were dissolved in 10 mL of MES buffer solution. Then 28 mg of EDC, 20 mg of NHS, and 38 mg of DA was sequentially introduced into the OHA solution. After 1 h of stirring in nitrogen atmosphere, the suspension was dialyzed against DI water for 3 d and freeze-dried at -80°C to obtain OHA-DA powders.

**Synthesis of adipic acid dihydrazide-modified hyaluronic acid (HA-ADH).** 1 g of HA powders were dissolved in 100 mL of MES solution. Then 1.25 g of EDC and 0.69 g of 1-hydroxybenzotriazole (HOBT) was introduced into the HA solution and reacted for 45 min, followed by adding 4.5 g of adipic acid dihydrazide (ADH) and reacting for 24 h. The suspension was dialyzed against DI water for 7 d and freeze-dried at -80°C to obtain HA-ADH powders.

**2. Results**

**Figure S1.** A) Chemical structure and B) 1H NMR spectra of CS, CSWY, and WYRGRL. C) The standard curve of WYRGRL at various concentration.

**Figure S2.** UV-Vis spectra of PDA, Fe, and PFe.

**Figure S3.** Cumulative release of FAM-140 from nanoparticles in PBS at 6.5.

**Figure S4.** TEM image of the nMSN@140-(PFe/CSWY)3 nanoparticles after 4 h of soak in PBS at pH 4.5.

**Figure S5.** A) SOD- and B) CAT-like activities of the nMSN nanoparticle, PDA, Fe, PFe, and CSWY.

**Figure S6.** A) Chemical structure and B) 1H NMR spectra of HA, OHA, OHA-DA, and HA-ADH.

**Figure S7.** SEM images of freeze-dried DHA hydrogel

**Figure S8.** EDS mapping of the nMSN@140-(PFe/CSWY)_3_/DHA hydrogel

**Figure S9.** Step-stain sweeps of the nMSN-(PFe/CSWY)_3_/DHA hydrogel.

**Figure S10.** The interfacial toughness of the hydrogels evaluated by a 180-degree peeling test.

**Figure S11.** Cumulative release of nanoparticles from hydrogel in PBS at pH 7.4 or 6.5.

**Figure S12.** In vitro degradation behavior of the nMSN@140-(PFe/CSWY)_3_/DHA hydrogel in pH 6.5 PBS containing hyaluronidase.

**Figure S13.** High-resolution fluorescence staining images indicating the cellular uptake of free FAM-miR-140 and FAM-miR-140-loaded nanoparticles.

**Figure S14.** *Ex vivo* cellular uptake of the nMSN-(PFe/CSWY)_3_ nanoparticles in neonatal femoral heads.

**Figure S15.** A) Intracellular DCF fluorescence images of chondrocytes following various treatments. B) Quantitative analysis of DCF fluorescence intensity.

**Figure S16.** Quantification analysis for SA-β-Gal staining *in vitro*. All data are represented as mean ± SD (n = 3). **p* < 0.05, ***p* < 0.01, ****p* < 0.001, and *****p* < 0.0001.

**Figure S17.** Quantification analysis for p21 staining *in vitro*. All data are represented as mean ± SD (n = 3). **p* < 0.05, ***p* < 0.01, ****p* < 0.001, and *****p* < 0.0001.

**Figure S18.** A) Immunofluorescence staining images and B) quantification analysis for p16 in TBHP-treated chondrocytes after treatments with various nanoparticles. All data are represented as mean ± SD (n = 3). **p* < 0.05, ***p* < 0.01, ****p* < 0.001, and *****p* < 0.0001.

**Figure S19.** Quantification analysis for ADAMTS-5 staining *in vitro*. All data are represented as mean ± SD (n ≥ 3). **p* < 0.05, ***p* < 0.01, ****p* < 0.001, and *****p* < 0.0001.

**Figure S20.** A) Immunofluorescence double staining images and B) quantification analysis for IL-β and TNF-α in TBHP-treated chondrocytes after treatments with various nanoparticles. All data are represented as mean ± SD (n = 3). **p* < 0.05, ***p* < 0.01, ****p* < 0.001, and *****p* < 0.0001.

**Figure S21.** A) Immunofluorescence staining images and B) quantification analysis for Col II in TBHP-treated chondrocytes after treatments with various nanoparticles. All data are represented as mean ± SD (n ≥ 3). **p* < 0.05, ***p* < 0.01, ****p* < 0.001, and *****p* < 0.0001.

**Figure S22.** Quantitative analysis of BV/TV and Tb.Th from the micro-CT results.

**Figure S23.** Quantification analysis for A) p21, B) IL-6, C) MMP-13, and D) Col II from immunofluorescence and immunochemical staining sections *in vivo*. All data are represented as mean ± SD (n = 3). **p* < 0.05, ***p* < 0.01, ****p* < 0.001, and *****p* < 0.0001.

**Figure S24.** High resolution image of GO enrichment analysis of the differentially expressed proteins.

**Figure S25.** High resolution image of chordal plots of KEGG enrichment analysis of the differentially expressed proteins.

**Figure S26.** High resolution image of GSEA enrichment analysis.

**Figure S27.** High resolution image of heat map indicating the differentially expressed proteins enriched in cellular senescence, NF-κB signaling pathway, p53 signaling pathway, and glycosaminoglycan degradation..

**Table S1.** Relative atomic percentages of each atom.

| Atomic (%) | nMSN | nMSN-(PFe/CSWY)_3_ |
| --- | --- | --- |
| Si | 26.8 | 15.6 |
| C | 14.8 | 37.6 |
| N | 3.9 | 4.7 |
| O | 54.3 | 41.0 |
| Fe | 0.2 | 1.1 |

**Table S2.** Primers for qRT-PCR.

| Gene | Sequence (5’-3’) | |
| --- | --- | --- |
| *GAPDH* | Forward | GAAGGTCGGTGTGAACGGAT |
|  | Reverse | CCCATTTGATGTTAGCGGGAT |
| *p21* | Forward | GAAAACGGAGGCAGACCAG |
|  | Reverse | TTCAGGGCTTTCTCTTGCAG |
| *IL-6* | Forward | ACAAGTCCGGAGAGGAGACT |
|  | Reverse | ACAGTGCATCATCGCTGT |
| *IL-1β* | Forward | TCCTCTGTGACTCGTGGGAT |
|  | Reverse | TCAGACAGCACGAGGCATTT |
| *MMP13* | Forward | GGACAAAGACTATCCCCGCC |
|  | Reverse | GGCATGACTCTCACAATGCG |
| Col2A1 | Forward | AGGGCAACAGCAGGTTCAC |
|  | Reverse | GCCCTATGTCCACACCAAATTC |
| ACAN | Forward | AACTTCTTCGGAGTGGGTGGT |
|  | Reverse | CAGGCTCTGAGACAGTGGGG |
